# The Visual Ecology of *Anolis* Lizards

**DOI:** 10.3390/ani16182848

**Published:** 2026-09-10

**Authors:** Leo J. Fleishman

**Affiliations:** Department of Biology, Union College, Schenectady, NY 12308, USA; fleishml@union.edu

**Keywords:** *Anolis*, dewlap, vision, retina, color, fovea, evolution, visual ecology, communication

## Abstract

Visual ecology explores how animal visual systems are related to light environments and important visual tasks. *Anolis* is a species-rich genus of small lizards that rely extensively on vision. Different species occupy habitats with different vegetation structure. The spectral properties of the habitats are similar, but total light intensities vary widely. They possess laterally positioned eyes with broad monocular visual fields, and a small region of binocular overlap toward the front. They possess a high-resolution central fovea associated with analysis of important images. There is a second, smaller, temporal fovea located where the visual fields of the two eyes overlap, which is associated with distance perception during prey capture. Color and brightness vision depends on four classes of single cones with different spectral absorbance curves, including one sensitive to the ultraviolet and a set of double cones. Most species studied possess similar sets of cones. Cone responses are modified by oil droplet filters, which are more variable among species and may play some role in adaptations to habitat light. Anoles communicate with a colorful expandable throat fan called the dewlap. Dewlap visibility depends on contrast with the natural background. Red dewlaps are most visible in bright, unshaded habitats. Yellow dewlaps are most visible in darker, shaded habitats. Visual displays include motion patterns of the head, body, and dewlap. The most highly visible movements are rapid up-and-down, start-and-stop patterns. These often occur at the beginning of visual displays and draw the attention of conspecifics to the displaying animal. Most *Anolis* species share similar visual-system properties. Differences in light environments interact with visual-system responses to influence the physical properties of communication displays and other visually-based behaviors.

## Simple Summary

Visual ecology is the study of how animal visual systems are related to the light, color, and motion of the world around them. *Anolis* is a species-rich genus of small lizards that rely extensively on vision. Different species occupy habitats that differ greatly in vegetation structure. These habitats differ little in color properties, but vary greatly in shade levels and in total light intensity. *Anolis* possess relatively large eyes that can perceive fine spatial detail. Most species share the same color-vision capabilities. They have excellent color vision that includes the ability to perceive ultraviolet light. They communicate using a colorful, expandable throat fan called the dewlap. Dewlap colors of different species vary with habitat. In brightly lit habitats, red stands out most strongly against the background of green vegetation, and most species have red dewlaps. In darker, more shaded habitats, yellow dewlaps are more visible against the green background and are most common. *Anolis* also respond strongly to certain visual motion patterns. They have evolved visual displays that utilize highly visible movements to attract the attention of other lizards. With their rich species diversity and high-quality visual systems, they have become an important model system for studies of vertebrate vision.

## 1. Introduction

Visual ecology is the study of the evolutionary relationship between the visual system of a species and the visual world in which the animal functions [1,2]. It includes the study of the visual tasks on which the animal’s survival and reproduction depend, the light conditions in which they occur, and the relationships between the physiology and anatomy of the eyes and relevant visual tasks.

Understanding evolutionary processes usually requires a comparative analysis. Two factors have to be considered. First, the shared common ancestry of the species in the group of species examined results in a shared set of ancestral traits. These inherited traits may represent a shared starting point from which diverse properties evolve, or they may represent a set of constraints that limit the extent of evolutionary divergence in response to different stimulus conditions. It is thus important to identify the shared ancestral traits and to identify those that are subject to change over evolutionary time, and those that are, for whatever reason, conserved and may act as constraints on evolutionary adaptation.

In order to understand the limiting effects of shared ancestry and the evolutionary diversifying effects of habitat and behavioral task differences, one ideally wants to study a large group of closely related species with known phylogenetic relationships, whose species operate in different light environments or whose visual systems perform different tasks. Anoline lizards are such a group. *Anolis* is a well-studied model system that has been used to gain insights into evolutionary processes in ecology, behavior, and physiology [3]. The anoles rely on vision as their primary sensory modality. They occupy habitats ranging from unshaded desert to heavily shaded closed-canopy forest. There are approximately 430 identified species [4] and their phylogenetic relationships, basic ecology, and behavior are well studied (reviewed in [5]). The anoles are well-established as an important model system for studies of terrestrial ecology, behavior, and evolution. Finally, their visual system and visually-based behaviors are relatively well-studied.

A useful feature of *Anolis* as a visual ecology model system is that all species share a critical visual task: communicating with visual displays consisting of motion patterns of the head, body, and a colorful, expandable throat fan known as the dewlap. Display motion patterns and dewlap colors differ across species, and it has been hypothesized that these colors and movements have evolved properties that strongly stimulate the visual systems [6]. Thus, diversity in visual function may have played an important role in the evolution of this among-species signal diversity, and the signal diversity is believed to have played a key role in driving the divergence into multiple species [5,7]. In this paper, I review our basic knowledge of *Anolis* vision and visual ecology and consider the relationship between visual signal properties—motion and color—and visual system function.

Here, I present a comparative overview of the visual ecology of *Anolis* lizards. All described species feed on small moving prey—usually small invertebrates such as insects and spiders. The species occupy distinctly different microhabitats due to specialized locomotion capabilities and thermal requirements [5,8,9]. These microhabitats differ in their light environments due to differences in shade level, typical height above the ground, the nature of background vegetation, and rainfall patterns [10]. Since many of the species are found in geographically separated locations—islands in many cases—there are phylogenetically distinct replicates of each type of microhabitat. For example, there are a number of deep-shade specialists, partial-shade specialists, unshaded-grass specialists, forest-canopy specialists, desert specialists, etc. In most cases, species from a given geographic area are more closely related to one another than they are to species from other localities that share habitat types. This allows us to ask questions about the relationship between visual system properties and signal evolution.

This paper is a review of published work on the visual ecology of anoles. The objectives of this paper are threefold. First, to provide an overview of basic anatomical, physiological, and behavioral features of the anoline visual system. Second, to examine the relationship between visual system features of different species and their habitat light conditions, looking to see if the species’ visual systems have diversified in response to differences in habitat light or other factors. Third is to examine the relationship between visual system properties and important visual tasks related to communication behavior: color perception (of the dewlap) and motion perception.

A number of the studies reviewed here have characterized and analyzed visual processing in anoles using behavioral, anatomical, genetic, and physiological methods, creating a strong base of knowledge regarding *Anolis* vision. These data can then be used to construct models of visual perception and have made it possible to explore the impact of differences in habitat light and visual stimulus variables beyond the direct measurements made in the lab. These models can be used to test the relative visibility of different visual stimuli under different light environments recorded in the field, in order to test the impact of visual perception and habitat light diversity on the evolution of signal differences among species.

This paper starts with a basic description of the structure and optics of the *Anolis* eye. This is followed by a comparative analysis of the anatomical and physiological properties of the retina, and then by a review of behavioral studies of responses to color, brightness, and motion. These studies provide quantitative assessments that allow us to predict the responses of the visual system to the different colors and motion patterns that comprise anoline displays. In this way, we can determine the role of visual physiology and habitat light in the evolution of signal diversity among species.

This manuscript is meant to be a complement to a recent review of lizard visual ecology [11]. There is some unavoidable overlap, since *Anolis* is one of the most studied examples of lizard vision. This paper, however, focuses in much more detail on *Anolis* and includes consideration of the relationship between its visual system and its signaling behavior.

## 2. Materials and Methods

In this paper, I use *Anolis* lizards as a model system to explore visual ecology in terrestrial habitats. The major focus of this review is on the research carried out by my collaborators and myself over the last 40 years. In addition, I review relevant literature on the topic from other authors. In the interest of brevity, not all topics and papers are covered. I have selected manuscripts to include based largely on my own familiarity with the topic. In addition, I conducted a basic literature search on Google Scholar using the keywords listed above for this paper. I searched the Web of Science database using the term “*Anolis*”. I searched the years 2000–present and identified a total of 205 references, only a small number of which were relevant for this paper. In the interest of brevity, I have, for the most part, restricted the review to work on *Anolis*, covering work on other species only when the results contribute directly to understanding of *Anolis* visual function. If readers are interested in a broader coverage of topics related to sensory biology and communication in lizards beyond just anoles, I would suggest looking at refs. [6,11].

## 3. The Anoline Eye

### 3.1. Basic Anatomy

**Figure 1:**
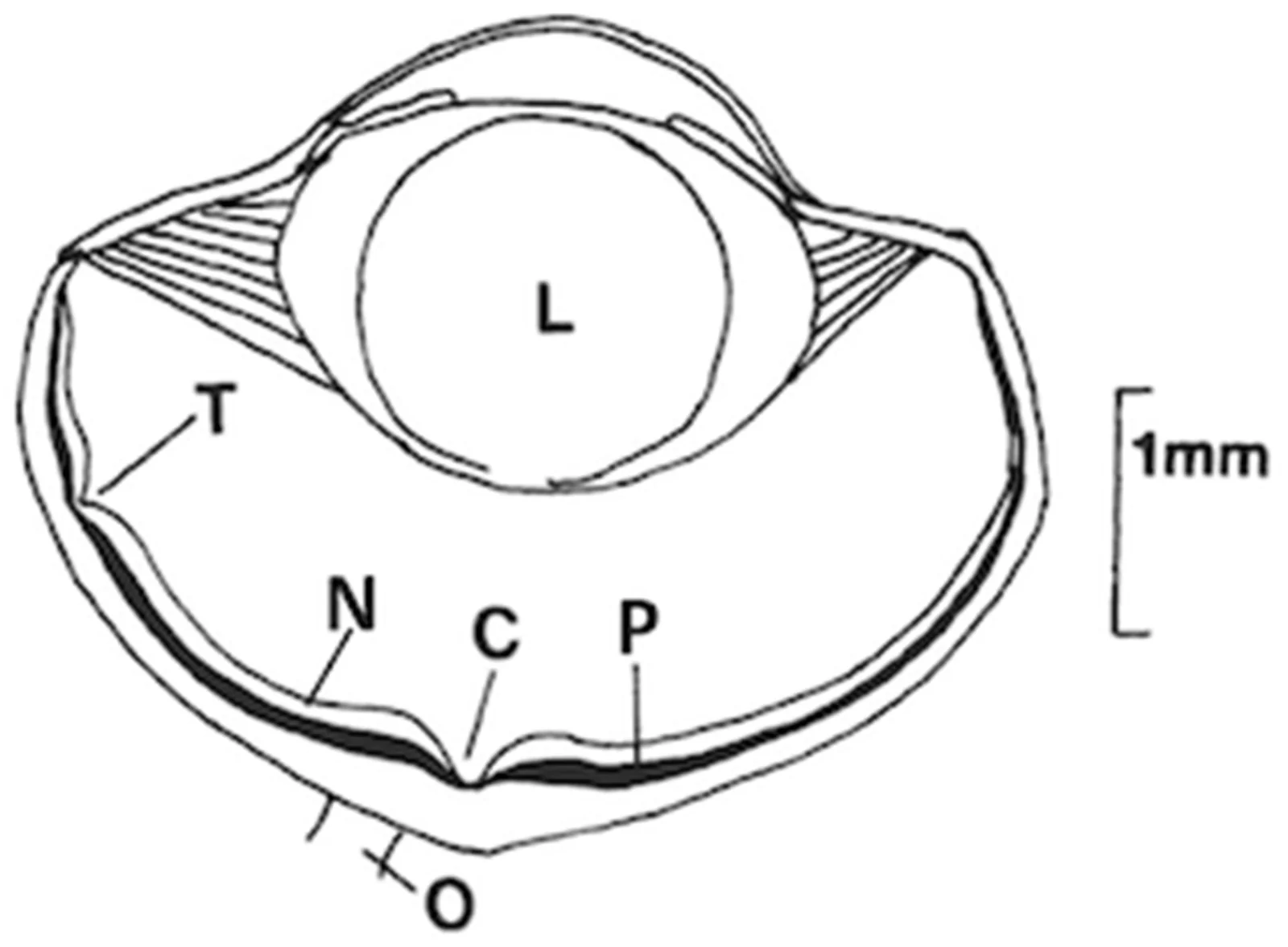
Top-down view of a cross section of the left eye of *Anolis lineatopus* with its nose pointed to the right. C, central (convexiclivate) fovea; L, lens; O, optic nerve; P, photoreceptor layer; N, neural layer; T, temporal fovea. The white area immediately in front of the photoreceptor layer is the neural layer that includes the ganglion cells that carry visual information to the brain. Reproduced with permission from G. Underwood, Biology the Reptilia Volume 2; published by Academic Press, 1970.

Basic anatomical techniques have been used to provide knowledge of the properties of anoline eyes [12,13,14,15,16]. Figure 1 presents a top-down view of a section of the eye, in the plane of the optic axis, of *Anolis lineatopus*, a medium-sized lizard native to Jamaica. The structure and shape of the eye are similar across most *Anolis* species. The two eyes are oriented nearly laterally on opposite sides of the head, angled slightly nasally. Each eye moves independently. Each eye can detect a visual angle of approximately 200 deg. There is a small zone of binocular overlap of the visual field, nasally (to the right in Figure 1). The eyes are relatively large for the size of the animals, and the two eyes nearly fill the head [12].

The lens is very flexible and is in direct contact with a muscle associated with the ciliary body. Contraction of the ciliary muscles directly bends the lens to change the focal distance to effect rapid, precise accommodation, in order to adjust and maintain the focus of objects at different distances. The curved cornea also contributes to focusing images on the retina [12].

The retina covers the entire inner region of the eye (opposite the cornea). The photoreceptor layer (P in Figure 1) contains the cone photoreceptors and the synapses they make with bipolar and horizontal cells. The cones are oriented with their long axis perpendicular to the retina. The pigment-containing outer segments are on the outside of the retina embedded in a pigment epithelium. Anoline retinas contain no anatomically identifiable rods [12,15]. Toward the cornea from the photoreceptor layers is a layer of neural and neuroglial elements that function to process the image and stimulate the ganglion cells. The ganglion cells form the optic nerve that carries visual information to the brain.

Light rays emanating from a point outside the eye pass through the pupil and are bent inward by the cornea and the lens to come to an approximate point (or, in reality, a small blur circle) on the retinal photoreceptor layer. The light rays pass through the neural layers of the retina and through the cone inner segments before reaching the visual pigments in the outer segments of the photoreceptors. Since light must pass through the neural layers, they are transparent. However, the index of refraction of the neural layers differs from that of the vitreous fluid that fills the eye. This difference becomes important when considering the role of the two foveal regions of the retina (below).

The cones are tightly packed together in the photoreceptor layer of the retina. The density of their spacing and their diameter ultimately determine the limit of spatial resolution. In those species that have been examined, the diameters of the thinnest visual photoreceptors in the center of the retina average approximately 1 µm [12]. This appears to be near the smallest size possible for photoreceptor cells that are still able to function [17]. In other parts of the retina—the visual periphery—the photoreceptor diameters are larger, reaching approximately 10 µm [12,13].

While most of the anatomical features of the eyes scale up in larger species, the size of the photoreceptors is fairly consistent across species. Larger eyes produce a larger image. Since the photoreceptor density does not increase with eye size, the capacity to detect fine detail increases with the size of the eye [11].

### 3.2. Spatial Acuity of the Eye

Spatial acuity is often measured in terms of “grating acuity”: the number of repetitions of black and white lines that can fit in one degree of visual angle, and still be perceived as separate lines. Acuity is determined by the size of the image that forms on the retina—which depends on the size and optical properties of the eye (which can be easily estimated—see [18]), the diameter of the individual photoreceptor cells, how densely they are packed into the retina, and the ratio of ganglion cells to photoreceptor cells. This quantity is important because in some visual systems the output of multiple photoreceptors converge on single ganglion cells, which increases sensitivity at the expense of spatial detail [19].

Makaretz and Levine {13] provided details of retinal anatomy for the medium-sized anole, *A. carolinensis*. The highest acuity region of the eye is the central fovea, where the narrowest and most densely packed photoreceptors are located, and the photoreceptor-to-ganglion cell ratio is less than 1. The estimated grating acuity for this part of the eye is 12–14 cycles per degree [18]. For comparison, the maximal human acuity is approximately 60 cycles deg^−1^ [2]. The lower value for the anoles is due to the small size of the eye, which results in a relatively small image on the retina. In the periphery of the retina, the grating acuity falls to 1.25 cycles per degree due to the larger diameters of the cones. Fleishman et al. [18] used a behavioral experiment to estimate the acuity in the visual periphery of a similar-sized species (*A. sagrei*) and determined an estimated grating acuity of 1.22 cycles deg^−1^, which is in good agreement with the calculated value for the similar-sized *A. carolinensis*.

### 3.3. The Central Fovea

The greatest density and smallest diameter photoreceptors occur in the central retina, which also exhibits a high ratio of ganglion cells to photoreceptors. This region is called the central fovea (see Figure 1). In this region, the neural layers that sit in front of the photoreceptor layer in the light path are shifted to the side to form a steep-walled funnel shape referred to as convexiclivate [12]. Fite and Lister [14] examined this structure in multiple species and found it to be similar in size and shape, even among species whose eye size was quite different.

The function of the convexiclivate central fovea is a matter of speculation. Three main hypotheses have been proposed. Because of the high density of photoreceptors in this region, the neural tissues that are stimulated by the photoreceptors are displaced to the side, forming the funnel shape of the fovea. The refractive index of this material differs from that of the fluid filling the interior of the eyeball. Light entering this region will be bent outward and enlarge the image falling on the photoreceptors. Walls [20] hypothesized that this causes a localized magnification of the image, enabling the ability to see finer detail of the image. Pumphrey [21] argued that this process will actually distort the image, making it harder to accurately detect fine details. He hypothesized that the central fovea is designed to enhance the detection of moving images, causing them to change shape and jump in an exaggerated manner as they move across the central fovea. Harkness and Bennet-Clark [22], studying chameleon eyes, proposed that the convexiclivate shape results in a patch of the retina that is highly sensitive to the small changes in focus caused by viewing objects at different distances. This would, in principle, provide an animal with an extremely sensitive depth-perception capacity, based on fine changes in focus. More recent studies have found some support for each of these hypothesized functions [23,24].

There is no direct evidence demonstrating the role of the convexiclivate fovea in anoles. It is worth noting, however, that a motion stimulus in the visual periphery causes a rapid shift of gaze that appears to place the image on the fovea. This suggests that increased perception of spatial detail is more important than detection of motion, since motion seems to be detected in the periphery and causes a shift of the image onto the central fovea. The high ratio of ganglion cells to photoreceptors [13] hints at some complexity of neural analysis of this region, as might be expected if the fovea is serving more than one function.

### 3.4. The Temporal Fovea

A unique feature of the anoline retina is the presence of two foveal regions. A second fovea is located in the temporal region of each eye (Figure 1) [12]. It is convexiclivate in shape but smaller and shallower than the central fovea. The density of photoreceptors here is higher than in other areas of the peripheral retina, but considerably lower than in the central fovea. Temporal foveas are found in the retinas of a number of bird species, but are not found in any other group of lizards. They lie in a small region of the eye where the visual fields of the two eyes overlap. They appear to play a critical role in prey capture. In a typical pre-capture sequence, an anole will detect a moving prey item and shift its gaze so the image lies on the central fovea of one eye. It will then move closer and abruptly shift its head position so that the prey item lies directly in front causing the image of the prey tp be positioned on the two temporal foveas [25]. The lizard then lunges forward to capture the prey in its mouth.

The actual role of the temporal foveas in this sequence has not been demonstrated. The two likeliest explanations are (1) that the positioning of the prey item simultaneously on the two foveas provides additional spatial detail about the food’s position and helps the lizard center its head position for an on-target lunge or (2) that the placement of the prey item simultaneously on the two eyes provides distance information that is improved in some way by the temporal foveae. For this, there are two likely possibilities. First, the convexiclivate shape of the fovea might make the eye sensitive to small changes in focus with distance, as has been argued for chameleon prey capture. Second, if the lizard positions the prey item simultaneously on the right and left temporal foveas, the extent of convergence of the two eyes needed to achieve this may provide an accurate distance cue. I am not aware of any published tests of these hypotheses. Since anoline eyelids move with their eyes, it should be possible to attach small pointers to the eyelids and observe whether or not the eyes converge as the animal gets closer to its prey.

### 3.5. Monocular Depth and Distance Perception

The greatest part of the visual field of each eye is monocular, and the two eyes move independently. This raises interesting questions about how they are able to perceive depth and distances while viewing the world monocularly. We know that they can judge distances at least over a range of several meters. Steinberg and Leal [26] carried out experiments (described later) in which tethered intruders were positioned at different distances from territorial males. The males adjusted the amplitude of their threat displays in order to create an optimal amplitude of image motion at the intruders’ eyes. This ability to accurately adjust display amplitudes depends on the ability to estimate the distance to the intruder. Henningsen and Irschick [27] showed that dewlap size is an indicator of fighting ability that competitors attend to. In order to judge the size of an intruder’s dewlap, they must be able to estimate how far away the competitor is. In addition, they need to judge distances to jump from perch to perch.

It is not known how anoles judge distance when viewing the world monocularly. We can suggest some possible (and non-exclusive) mechanisms. For short-range distance estimation (e.g., 1–2 body lengths away), they might rely on focus adjustments to measure distances, as has been shown for chameleons [28]. It has been argued that the convexiclivate fovea causes images to quickly go in and out of focus with distance, which improves the animals’ capacity to judge distances in this way [22]. It is conceivable that anoles have some capacity to judge distance with changes in focus of images on the convexiclivate fovea. However, they lack several of the optical specializations observed in chameleons, and their eyes are much smaller, which means changes in distance will have a smaller effect on focus.

The mechanisms described will not be useful for judging distance over longer ranges. One possible mechanism is motion parallax, where small head movements cause images of nearby objects to move by different amounts and in different directions than more distant objects. Anoles frequently make small rapid up-and-down head movements, and it has been hypothesized that these might generate parallax cues [29,30]. Other likely distance cues are the relative size of known objects, the tendency for distant objects to appear smaller, linear perspective, and the position of objects in front of or behind others [31,32]. 

## 4. Habitat Light

A critical question in any study of visual ecology is whether different species inhabit distinctly different light environments. It is well established that different anoline species occupy different microhabitats [5,8]. The next question is whether these different habitats result in different light regimes.

Two variables are most useful for characterizing habitat light environments: side-welling irradiance and side-welling radiance [33]. Irradiance is a measurement of the hemisphere of light arriving at and illuminating a small flat surface. Its units are micromoles of photons per square meter per second (µmol m^−2^ s^−1^). To measure side-welling irradiance, a diffuse irradiance detector probe is oriented parallel to the ground, because the lizard’s eyes are generally oriented with their gaze directed parallel to the ground, and the dewlap, when expanded, is oriented perpendicular to the ground, and each side captures light from a full hemisphere. Irradiance thus measures the light that is typically illuminating the eye or a flat surface such as a dewlap. The background light at any given location is characterized by radiance (units are micromoles of photons per meter squared per second per steradian of solid angle, or µmol m^−2^ s^−1^ sr^−1^). This represents the light emanating from a small visual angle. It is used to measure the light emanating from the surface of an object (such as a leaf, tree trunk, or dewlap), or from a small patch of natural background (e.g., a sunspot). When measured repeatedly in the field, it captures the average and variability of the patches that make up the natural visual background. It is measured by replacing the irradiance probe with a small lens that collects light from a 4 deg solid angle. Irradiance or radiance can be measured in terms of the total light collected over time over the full range of visible wavelengths (intensity) or as spectral irradiance or radiance, which characterizes the light in terms of spectral content.

**Figure 2:**
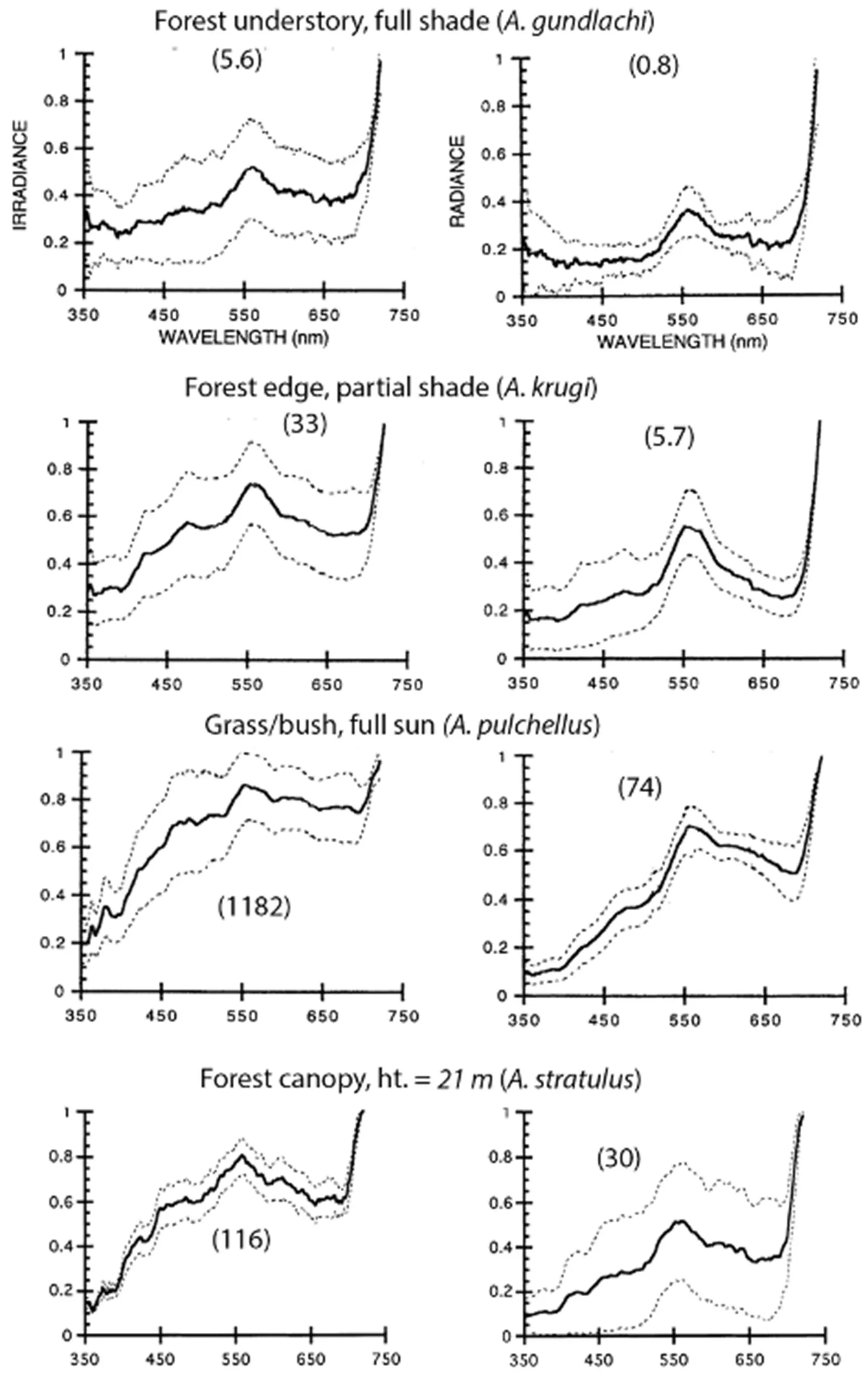
Measurements of habitat light (spectral quality and intensity) for four Puerto Rican species [36]. Samples of side-welling spectral irradiance and radiance were taken for these four species under clear skies at locations where lizards were observed. These represent typical habitats for many different anoline species. The units of irradiance are µmol m^−2^ s^−1^. Units of radiance are µmol m^−2^ s^−1^ sr^−1^. Data shown in the graphs are averages of normalized curves to illustrate the shape of the curves. Numbers in parentheses are average total intensity for each of these samples. Dotted lines indicate ±1 standard deviation. Reproduced with permission from Fleishman et al., J. Comp. Physiol A; published by Springer, 1997.

Figure 2 illustrates average normalized spectral irradiance (first column) and spectral radiance (second column) from four typical anoline habitats on Puerto Rico [34]. The first three habitats, which are typical for many different species, represent full shade, partial shade, and full sun (grassy areas with little shade). Measurements were taken at specific locations where lizards were directly observed in the field. The curves were normalized (equal area under the curves) and then averaged in order to illustrate typical spectral shapes. The average total intensity at these measured locations (=total irradiance or radiance, non-normalized) is shown in parentheses on each graph. The fourth dataset was collected from the top of a forest canopy tower, which is where the species *A. stratulus* is most common [35].

A few general conclusions can be drawn from these graphs. The first column illustrates spectral and total irradiance. The spectrum in the forest-shade habitat has a sharp peak at 560 nm. Moving down the column, the partial-shade habitat has a broader spectrum, with a smaller peak at 560 nm. In the open habitat, the shape of the spectrum shifts to one with a broad peak that resembles a spectrum of sunlight. The top of the forest canopy (*A. stratulus*) has a spectrum similar to partial shade but with greater intensity.

Radiance represents samples of the habitat background. In all four cases, the average has a clear peak at 560 nm. The overall spectrum broadens somewhat in the less shaded habitats (unshaded grass and canopy).

The most dramatic differences across the habitats, in both radiance and irradiance, are the differences in total intensity, with an increase by a factor of 10–100 or more with each shift to a less shaded habitat. Other studies have revealed that the differences in habitat intensity described here, based on shade preference, are fairly consistent when measured at different localities [36]. The measurements for Figure 2 were made under clear skies. The presence of cloud cover causes a modest reduction in intensity but does not change the spectral pattern [34].

## 5. Evidence of Adaptation to Habitat Light Intensity

Since the most dramatic difference in habitat light experienced by the different species is total intensity, one might expect to find evolutionary adaptations of the visual system to these differences. Fleishman et al. [36] tested for this possibility in three Puerto Rican species: *A. gundlachi* (forest shade), *A. cristatellus* (partial shade), and *A. pulchellus* (full sun) by measuring the response of the eyes to flickering light with electroretinography (ERG). ERG is a measurement of the electrical response of the eye to flashes of light measured with a wire touching the surface of the eye (see description below). The frequency of flashed light at which there is no longer a response to each flash is called the critical frequency of fusion (CFF). The faster the eye can respond, the higher the CFF. It was hypothesized, for example, that full-sun species would exhibit a higher CFF in order to take advantage of the higher light intensity for rapid motion detection. However, it was found that the CFF values, and the overall response to flicker at different frequencies, of the three species were nearly the same. In a second study, Fleishman et al. [34] compared the same three species’ behavioral response when placed inside a rotating striped drum. When the stripes on the drum are visible, this stimulus triggers a reflexive optomotor response in which the head and eyes turn to follow the rotating stripes. They determined the minimum light intensity for each species that triggered the behavioral response. There was no significant difference in the intensity required to elicit the behavior in the full-shade and partial-shade species. *Anolis pulchellus*, the full sun species, required a significantly higher stimulus intensity to elicit the response. The physical basis for this difference is untested. It may be related to the size of the different species. *A. pulchellus* is smaller and has smaller eyes and pupils than the other two species. A smaller eye creates a smaller image on the retina and, since photoreceptor cell diameters are similar in species of different size, the smaller image may limit the extent of spatial summation across the retina.

## 6. Retinal Photoreceptors

### 6.1. Cones and Their Pigments

One of the fundamental questions in visual ecology is whether the eyes of a given species are evolutionarily adapted to the habitat conditions in which they exist. If this is the case, and closely related species occupy different light environments, we should detect differences in their visual physiology or anatomy that are related to differences in their habitat light. Here, we review the spectral response of the retinal photoreceptors of different species to see if they differ based on differences in habitat light.

Quanta of light are captured and translated into neural signals by the visual photoreceptors of the retina. Based on the anatomy of the photoreceptors, anoles have a retina that contains only cones and no rods. Their capacity to perceive color is based on the presence of different types of cones that have different visual pigments with different spectral sensitivities in their outer segments. Thus, each type of cone will respond to a different extent to any given spectral distribution of light in a visual stimulus. There are two main types of cones in the anoline retina: single and double. Double cones consist of an attached pair of cells—a thicker axial cell and a more slender peripheral cell [12,15]. Both members of the pair have the same visual pigment. All cones, except for the axial cell of the double cone, also possess an oil droplet located within the inner segment that filters the light traveling to the outer segment where the visual pigment is located. The axial member of the double cone lacks an oil droplet, but contains a dispersed pigment that is similar in its color filtering properties to the oil droplets.

Loew et al. [15] used microspectrophotometry (MSP) to measure the relative spectral absorbance (or relative spectral sensitivity) of the visual pigments in the cone outer segments, oil droplets, and diffuse pigment of the double cone axial member from 17 species of anoles. In MSP, the retina is isolated, removed, and spread out flat. An individual cone outer segment (or an isolated oil droplet) is exposed, and a narrow beam of light is passed through it. The change in intensity of the light as it passes through the cell is measured as the spectrum is shifted from 340 to 700 nm. In this way, the spectral absorbances (relative transmission) of individual cell elements are measured.

Visual pigments are typically defined by their wavelength of peak absorbance, λ_max_. Loew et al. [15] examined species from seven different clades that occupied a full range of habitat types, including fully shaded forest, partial shade (forest edge or dry forest), and unshaded grassland.

**Figure 3:**
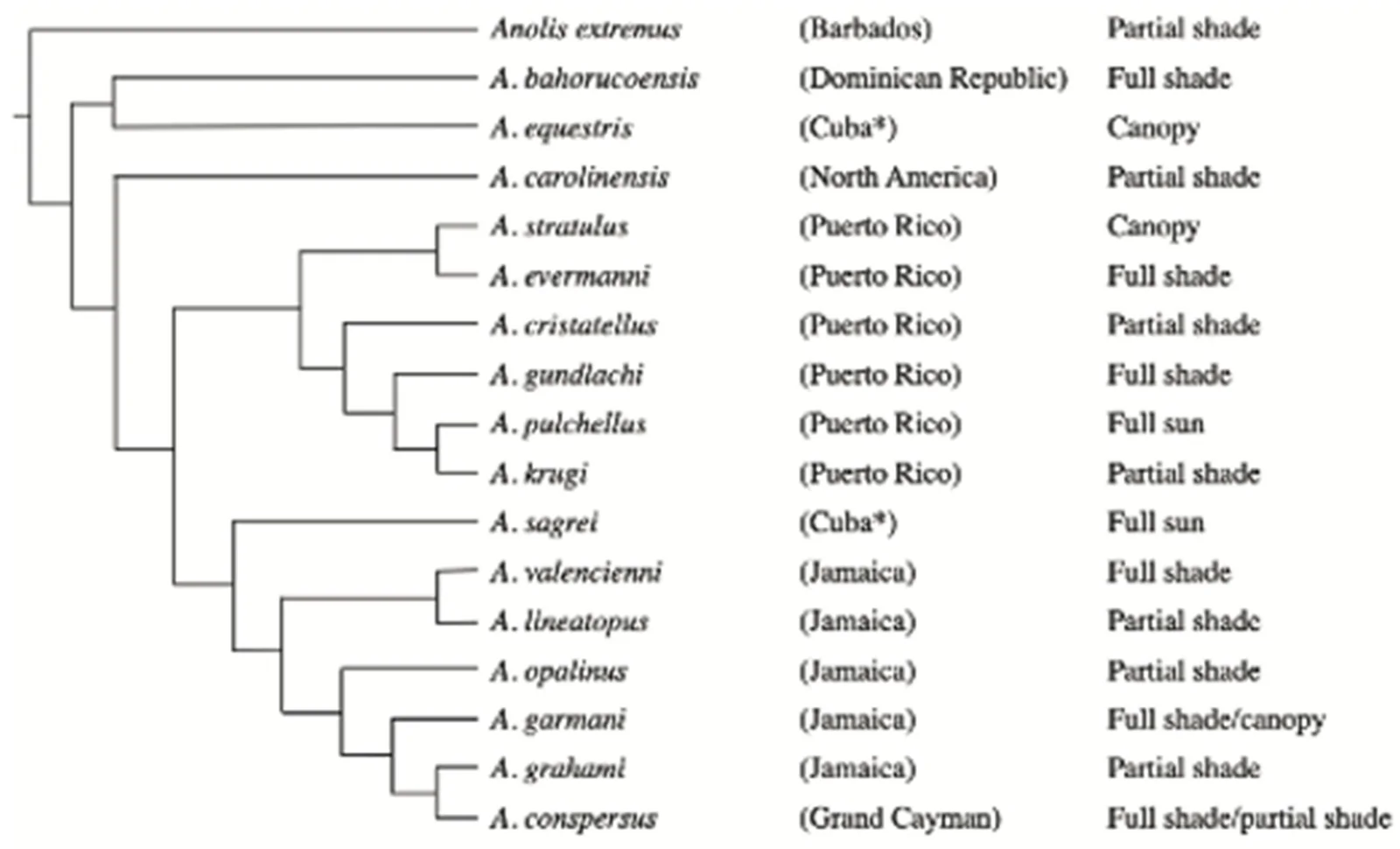
A cladogram, based on [37], showing the relationships among the species from [15]. The lengths of arms on the cladogram have no quantitative meaning. There are additional species between those listed in this cladogram. [37] identified 17 major distinct clades within the genus *Anolis*. Five of these are represented in this sample. * *A. sagrei* and *A. equestris* were collected from introduced populations in Florida and are Cuban in origin. Reproduced with permission from Loew et al., J. Exp. Biol.; published by The Company of Biologists, 2002.

In nearly all of the species studied, four cone classes with four distinct visual pigments were found. For a small number of species, only three pigments were found, but it was probable that the fourth pigment was present and simply not sampled because these cones are fairly rare in some retinas. Figure 3 summarizes the species that were examined, their evolutionary relationships, and the typical habitat of each.

**Figure 4:**
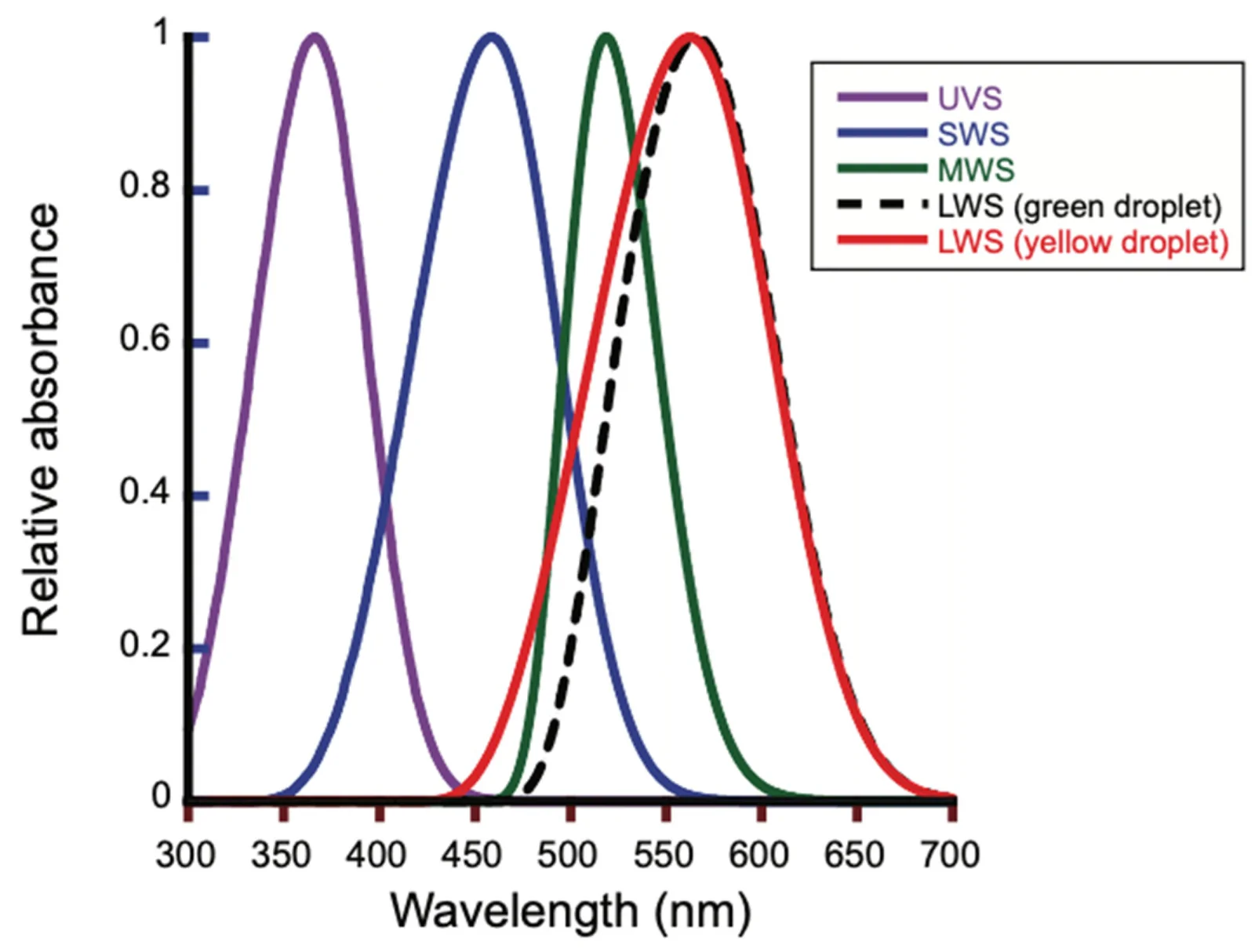
Absorbance spectra (normalized to a peak of 1.0) of four classes of cones (from *A. cristatellus*) modified by the oil droplet found in each cone type. The LWS cone comes with two different oil droplets (green (G) and yellow (Y)). The LWS/G oil droplet combination is indicated by a black dashed line. The LWS/Y oil droplet combination is shown in red. The shape of the spectral sensitivity curve of the double cones is the same as the LWS single cone with a yellow oil droplet. These spectra were calculated using Lamb’s pigment template [38]. The effects of the oil droplet were modeled using the method described in [39].

Figure 4 illustrates the normalized spectral sensitivities (absorbance spectra) of the four classes of photoreceptor outer segments found in the majority of species. Typically, cones were found with peak spectral sensitivity in the ultraviolet range (UVS, or ultraviolet-sensitive), in the short-wavelength (blue) range (SWS, or short-wavelength-sensitive), in the middle-wavelength range (MWS, or middle-wavelength-sensitive), and in the long-wavelength range (LWS, or long-wavelength-sensitive).

**Figure 5:**
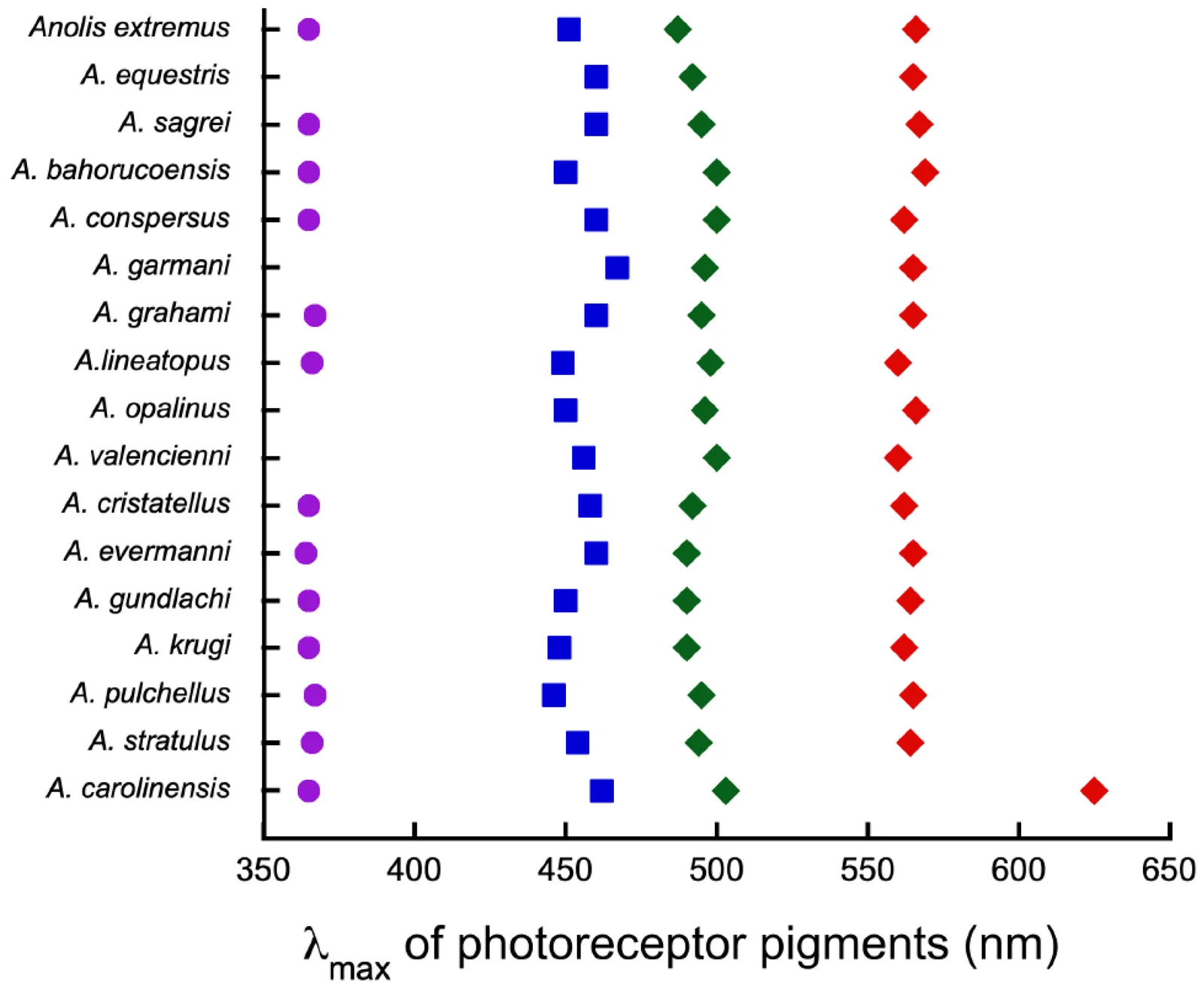
Wavelengths of peak absorbance of cone photoreceptor pigments in 17 species sampled in [15], listed in Figure 3. Most species were found to have four pigments. In a few cases, one of the four was not found due to sampling limitations. Reproduced with permission from L. Fleishman, Frontiers in Amphibian and Reptile Science; published by Frontiers, 2024.

The average absorption peaks of the visual pigments of all species are shown in Figure 5. In all but one case (see below), the average λ_max_ values were nearly the same. Loew et al. [15] detected no relationship between habitat light conditions and photoreceptor pigment absorbance maxima. There is some among-species variation in λ_max_ of the SWS cone. However, Lind et al. [40] modeled the effects of small variations in single-cone λ_max_ values. In birds (which have a set of cones similar to lizards), they found that there was very little effect on color perception of small variations in λ_max_ of individual photoreceptor classes.

One species, *Anolis carolinensis* (the last species listed in Figure 5), was found to have pigment spectral sensitivity peaks that were shifted to longer wavelengths. This is most evident for the LWS cone. There is no obvious ecological correlate, variation in behavior, or explanation for this difference. It is due to a difference in the molecular structure of the visual pigments. The *A. carolinensis* visual pigments are based on a vitamin A_2_ chromophore, whereas the other species sampled have pigments based on vitamin A_1_. This difference is known to shift the peak sensitivity of pigments to longer wavelengths [41].

The genetic code for each of the cone pigments has been identified, and it is the same as those found in other reptiles, birds, and many fish lineages. They are identified as SWS1 (for the UVS cone), SWS2 (for the SWS cone), RH2 (for the MWS cone), and SWS (for the SWS cone). Surprisingly, a second gene has been found that also codes for the MWS cone pigment, called RH1. In many non-lizard vertebrate species, RH1 codes for the pigment in rods. The role of RH1 in the anoline retina, and the significance of there being two different genes for MWS cone pigments are unknown [11,42].

### 6.2. Oil Droplets

All cone types except for the axial cones of the double cone pair possess an oil droplet. They act as long-pass optical filters and modify the spectral responses of their cones. They absorb, nearly completely, short-wavelength light up to a certain wavelength, and then shift abruptly to full transmission of the longer wavelengths. The wavelength at which transmission is halfway between minimum and maximum is referred to as λ_cut-on_. Four distinct classes of oil droplets have been found in the anoline retina. They are typically described based on their color appearance in light microscopy. Yellow oil droplets (Y) are found in some LWS single cones and all of the peripheral double cones. Green oil droplets (G) are found in some LWS single cones and in all MWS cones. There are two types of colorless oil droplets: C1 and C2. C1 are found in all of the SWS cones and absorb UV and violet light. C2 are found in all of the UVS cones. Loew et al. [15] did not measure transmission below 350 nm, but it is presumed that the C2 oil droplets absorb short wavelengths up to approximately 340 nm. The average cut-on wavelengths of the different oil droplets are summarized in Table 1.

Four typical combinations of pigment plus oil droplet absorption are shown in Figure 4. In each case, oil droplets remove most of the short-wavelength portion of the absorbance curve of the cone they interact with. It is believed that this filtering out of shorter wavelengths makes images less noisy, since short-wavelength light tends to scatter strongly. It also sharpens wavelength discrimination by making the sensitivity curves steeper on the short-wavelength side [15], and therefore makes cones more sensitive to small differences in color. The G oil droplet combined with the MWS cones creates a very steep sensitivity curve and shifts the λ_max_ value of the cone to a longer wavelength.

Since LWS cones are combined with either green or yellow droplets, two absorbance curves are possible for LWS cones. As shown in Figure 4, the curves are similar, and the peak sensitivity is the same in both conditions. In behavioral tests (described below), it was found that chromatic perception behavior could be accurately modeled assuming four cone channels, suggesting that the difference between LWS cones with the different oil droplets has only a small impact color perception.

Campbell and Loew [16] compared the frequency of yellow and green oil droplets in the retinas of three species. They found them to differ significantly: G droplets were most common in one species, while Y droplets were most common in the others. They suggested that these differences in oil droplet composition of the retina might represent adaptations to differences in the habitat light conditions of the tested species. However, the connection between habitat light and oil droplet frequency was unclear.

We cannot rule out the possibility that the presence of cones with the same pigment paired with different oil droplets represents two distinct perceptual channels. This might introduce an extra level of complexity in the color perception of anoles. These small changes might, for example, result in improved discrimination of fine differences in color, which could play a role in mate choice, where small variations in conspecific color can signal fitness differences. Examination of the role of oil droplets in lizard vision represents a challenging and exciting future area of study.

### 6.3. Single vs. Double Cones

Diurnal birds have photoreceptors and pigments that are very similar to those of diurnal lizards. In birds, it has been established that the four types of single cones contribute to chromatic sensation. The double cones, which possess the LWS pigment, are believed to be responsible for achromatic vision. Achromatic vision controls the sensation of brightness (a.k.a. luminance) and is the basis for perception of motion and spatial pattern [43]. It is possible that the double cones in the anoline retina similarly control achromatic sensation. It will be shown below that the spectral sensitivity of the LWS cones matches the achromatic sensation in anoles.

### 6.4. General Conclusions from Microspectrophotometry

In regard to visual ecology, we find that species from different clades living under very different light conditions share nearly the same retinal properties. The similarity of the photoreceptor spectral sensitivities for most species is likely due to their shared ancestry and the effectiveness of this shared color vision system across a variety of light environments. Osorio [44] described the visual pigments found in the retinas of diurnal birds and reptiles and found that (1) birds and reptiles share similar sets of cone pigments, and (2) in both birds and reptiles the set of cone pigments is very similar in most species. He also notes that these pigment types are genetically the same as those found in many fish species. In the fishes, there have been many evolutionary shifts in the spectral response of these cone pigments. This suggests that the relative constancy of photoreceptor pigment spectra in terrestrial birds and reptiles is not due to some strong genetic constraint. Rather, he suggests that the complement of photoreceptors found in these species is highly effective in a wide range of terrestrial habitats and a form of stabilizing selection has maintained them through evolutionary time.

## 7. Electroretinography

Another useful tool for studying the properties of the retina is the electroretinogram (ERG). In this method, an electrode is placed on the surface of the eye of an anaesthetized animal. Light of different wavelengths is flashed at the eye, and the strength of the response to these flashes, relative to an alternately flashed white control light, is measured. The technique yields a precise measurement of the overall spectral sensitivity of the eye [34]. ERG measurements of response to different wavelengths are useful for two reasons. First, the shape of the spectral sensitivity curve measured in this way depends on the relative abundance of the individual classes of cones present in the retina (modified by any oil droplet or other intra-ocular filters), and can therefore be used to detect large differences among species in the relative numbers of different types of cones. Second, human studies have shown that the ERG response provides a good approximation of the relative perceived brightness of stimuli of different wavelengths [45,46]. The relationship between wavelength of light and perceived intensity is referred to as luminance.

**Figure 6:**
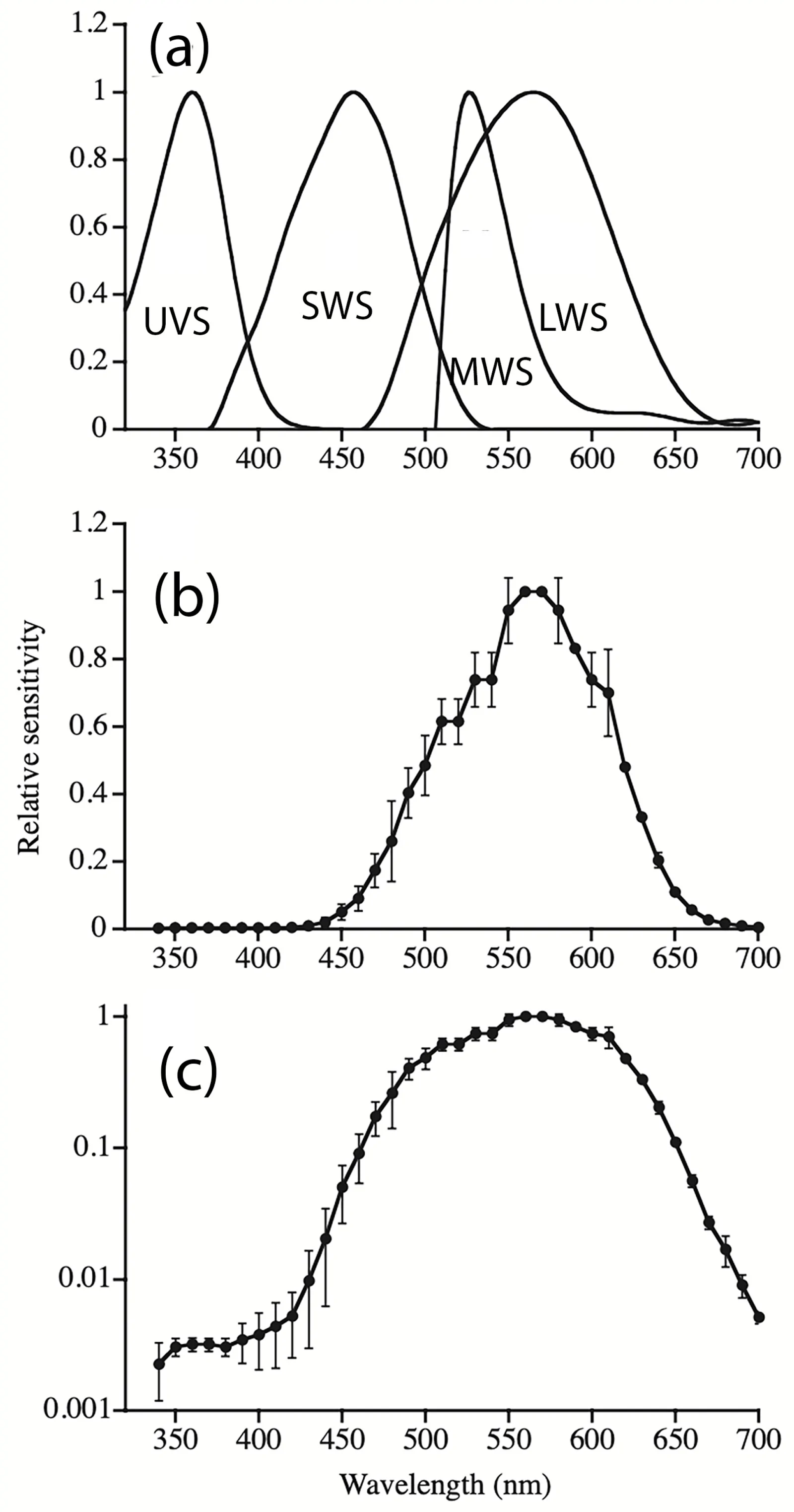
(**a**) The spectral sensitivity function for each of four classes of cones (including oil droplets) found in the retina of *Anolis cristatellus*. Cone types are ultraviolet-sensitive (UVS), short-wavelength-sensitive (SWS), middle-wavelength-sensitive (MWS), and long-wavelength-sensitive (LWS). (**b**) The overall spectral sensitivity of *Anolis cristatellus* based on electroretinography (ERG) at a stimulation rate of 6 Hz. The data are plotted on a linear scale for comparison with the curves in (**a**). (**c**) The ERG spectral sensitivity curve is plotted on a logarithmic scale to illustrate the small but measurable response at wavelengths less than 430 nm. Values are means ± S.D. *N* = 3. Reproduced with permission from L. Fleishman and M. Persons, J. Exp. Biol.; published by The Company of Biologists, 2001.

Fleishman et al. [34] measured ERG-based spectral sensitivity in six species of anoline lizards. In all six species, the overall spectral sensitivity (i.e., luminance) closely matched the spectral sensitivity of the LWS cones, with a peak in sensitivity near 560 nm. An example of the ERG-determined response in comparison to the LWS photoreceptor spectral response is shown in Figure 6. It is apparent from this figure that the perception of brightness (i.e., luminance) is largely a function of the response of LWS cones, and these cones are by far the most common type in the retina. In fact, the shape of the ERG-determined luminance function and the LWS cone spectral sensitivity is so similar that either curve can be used to estimate the relative luminance of different spectral stimuli. Further supporting the result that the ERG-based spectral sensitivity curve depends on the LWS cone pigment, Fleishman [47] measured the curve in *A. carolinensis*, which has red-shifted cone pigments. The ERG curve peak and overall spectral shape closely matched that of the red-shifted LWS cones.

The fact that, in the six species measured, the peak of the ERG-measured luminance response is 560 nm may be a convergent adaptive response to the shared property of habitat light. Achromatic perceptual functions such as motion or spatial detail perception are believed to be optimal when the overall spectral sensitivity of the visual system matches the average habitat radiance [1] which appears to be the case for the six species studied in ref. [34].

## 8. Behavioral Studies of Color and Brightness Perception in Anoles

### 8.1. Tests of Chromatic and Luminance Contrast

Determining the ability of an animal to perceive color typically requires tests based on behavioral conditioning [48]. Unequivocal proof of color perception, independent of luminance-based discrimination, requires a demonstration that an animal can be trained to select a given color even when presented over a range of different luminance values. This procedure has proven to be difficult with lizards, and it has never been accomplished with *Anolis*. Leal and Powell [49] trained *A. evermanni* to choose between two differently colored pegs covering wells in which food items were hidden. Lizards quickly learned to choose the correct colored cover. They learned quickly to discriminate between blue and yellow discs and between a solid color disc and one with colored rings. When the rewarded disc pattern was reversed, they rapidly learned to select the new rewarded disc. These experiments showed that anoles could learn to associate particular patterns and/or colors with food, which would be necessary for an unambiguous demonstration of color discrimination capabilities. They did not, however, demonstrate color discrimination capacity. The color choices differed considerably in luminance and/or pattern, so it was not clear whether their discrimination was based on luminance or spatial pattern differences rather than color.

Another approach has proven useful for the study of color perception in lizards based on a behavior known as the “visual grasp reflex”. This is a reflex shift of gaze direction that occurs when an image of potential interest, such as a prey item, is detected on the peripheral retina. This type of stimulus sometimes elicits a rapid shift in the eye position that places the image on the central fovea. The behavior is easy to observe because the eyelids move with the eye. In the experiments described below, a colored stimulus is moved into view in the visual periphery. The experimenter carefully observes whether or not the lizard abruptly shifts its gaze toward the new stimulus. The probability of a gaze shift toward the newly presented stimulus is quantified as a function of the tested stimulus variable.

Here, I describe three behavioral studies that were designed to quantify detection probability as a function of the contrast in color and brightness between a small stimulus flag and a colored and/or patterned background [43,50,51]. In each of these studies, subject lizards were isolated in a cage and sat on a perch parallel to the front wall. The front of the enclosure, directly opposite the direct monocular gaze, was clear, and a camera monitored the position of the outward-looking eye. In each experiment, a relatively large, colored background surface was positioned 45 degrees from the original gaze direction. In each trial, a small colored flag (approximately square-shaped and the size of a dewlap) was brought rapidly into view at the center of the background, held in position for a few seconds, and then moved out of view. If the lizard noticed the appearance of the stimulus, it rapidly shifted its gaze toward it. In each experiment, these trials were repeated many times with a number of different individuals to yield a probability of detection associated with each stimulus/background combination.

The first of these experiments was carried out on *A. cristatellus*. Lizards were presented with a uniformly illuminated background (either white or green, equal in luminance). In each trial, a small, colored stimulus flag was abruptly introduced into the visual periphery, in the center of the background area, and after a few seconds moved out of view. Four different stimulus spectra were tested, each presented at eight different luminance values. Stimulus luminance values were calculated by multiplying the ERG-based spectral luminance function (see Figure 6) by each stimulus.

Presentation of each combination of stimulus color and luminance against each background was repeated with approximately 40 individuals, and the probability of detection for each stimulus condition was determined.

**Figure 7:**
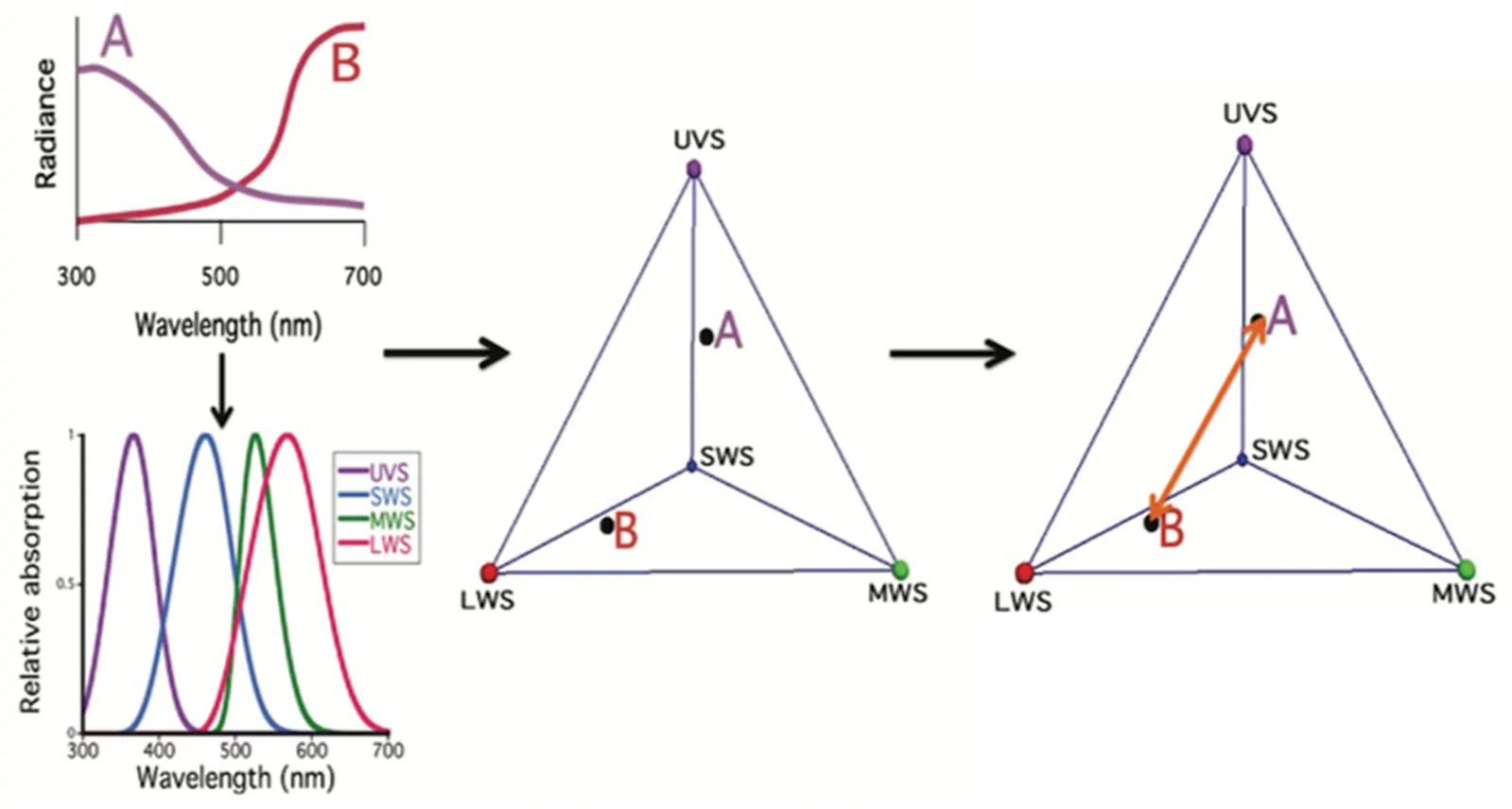
Chromatic contrast can be quantified as a distance in a tetrahedral color space. Each vertex of the tetrahedron represents one of the cone classes. The cone (plus oil droplet) absorbance functions are normalized to be equal in area under prevailing light conditions (typically side-welling irradiance). For any spectral stimulus, the relative absorbance of each of the four classes of cones is determined and divided by the total value for all four. A vertex of the tetrahedron represents a value of 1.0 (complete stimulation of one cone type, and none of the others) while the opposite side of the tetrahedron indicates a value of zero. After two spectra have been quantified in this way, a Euclidean distance between them can be calculated, as illustrated by the orange arrow connecting A and B in the pyramid on the right. This distance provides a quantitative measure of the perceptual distance between the two spectra. This value can be used, for example, to quantify chromatic contrast between a colored stimulus and the background against which it is viewed, as in [43].

For the analysis of the results, stimulus/background interactions were quantified with two variables: luminance contrast and chromatic contrast. Luminance contrast (C_L_) was defined as (L_S_ − L_B_)/(L_S_ + L_B_), where L_S_ = stimulus luminance and L_B_ = background luminance. Notice that a stimulus darker than the background yields a negative value. Chromatic contrast (C_C_) was defined as a distance in chromatic sensory space. This value was based on a geometric distance between two spectral stimuli plotted in a tetrahedral color space (see Figure 7). It represents the difference in the relative stimulation of four cone classes (plus oil droplet filters) in response to the background spectrum vs. the stimulus spectrum (details in [43,50]).

**Figure 8:**
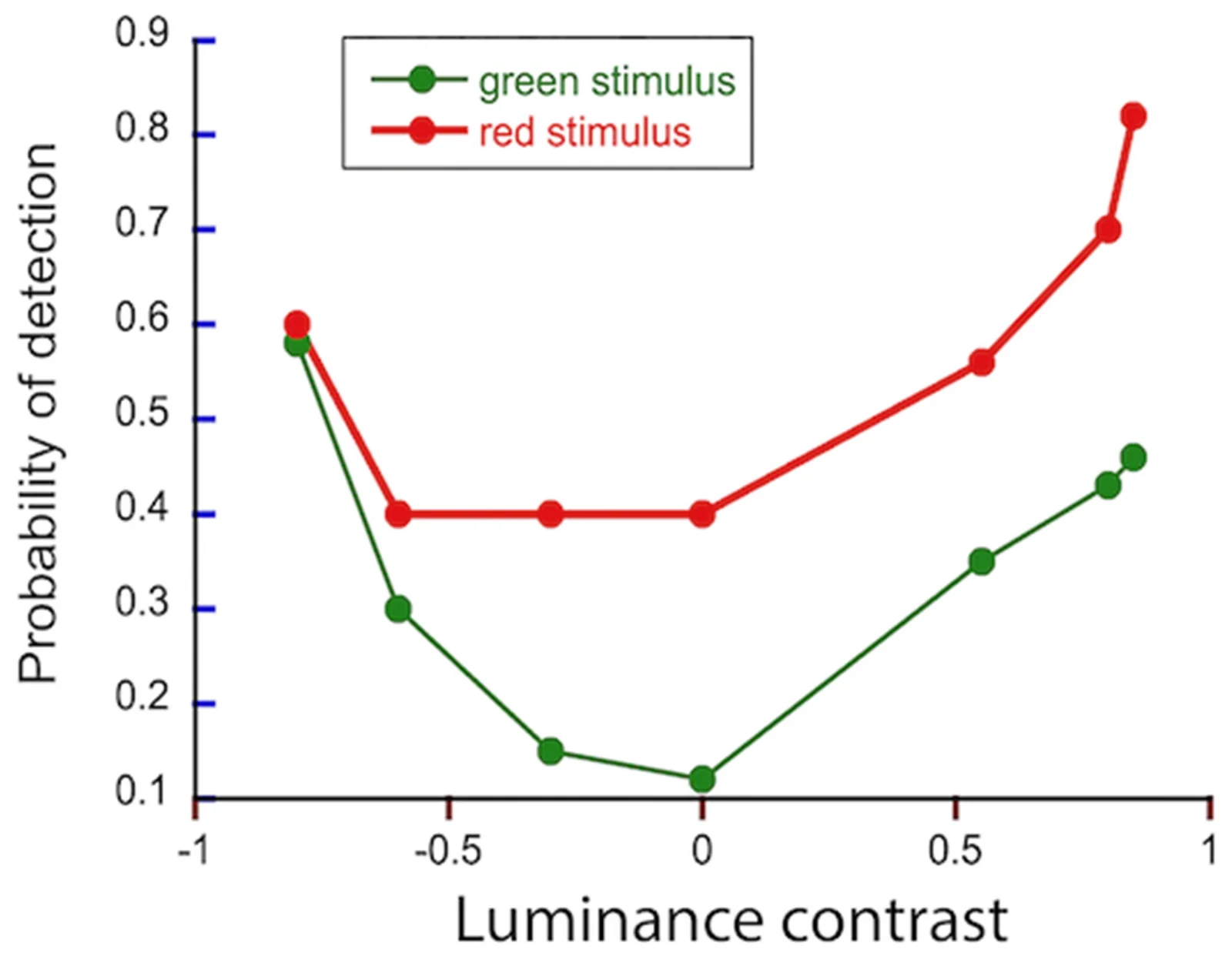
Example of two sets of results from [43]. Colored stimulus flags of differing spectra (red or green in this case) and luminance were introduced in front of a uniformly covered background (green in this example). Probability of detection for each stimulus/background combination in these experiments is shown. Typically, the lowest response occurred when the stimulus and background had nearly the same luminance. The response probability increased in a V-shaped pattern as luminance contrast increased. Luminance contrast could be either positive (brighter than background) or negative (darker than background). Increased chromatic contrast elevated the response probability across all luminance contrast values, shifting the V-shaped curve upward. Four stimulus spectra and two background spectra were tested in the experiment. Reproduced with permission from L. Fleishman and M. Persons, J. Exp. Biol.; published by The Company of Biologists, 2001.

An example of one set of results is shown in Figure 8. In general, response probabilities to each stimulus color were V-shaped. The smallest response occurred at the point where stimulus and background luminance were nearly the same, and response increased linearly with increasing positive or negative luminance contrast. The entire set of V-shaped responses shifted upward when chromatic contrast was increased by changing stimulus or background color.

These experiments showed that the probability of detection was a linear additive combination of the chromatic contrast (C_C_) plus the absolute value of luminance contrast (C_L_). Fleishman and Persons [43] carried out a multilinear regression on response probability versus luminance contrast and chromatic contrast and concluded that detection probability (P) = 0.40|C_L_| + 0.43C_C_ + 0.16. The regression had an R^2^ value of 0.7 and explained a highly significant portion of the variation in the data (*p* < 0.0001).

These results indicated that detection probability of colored stimuli against colored backgrounds could be predicted based on the assumption that anoline color vision is based on the relative stimulation of four classes of single cones plus the luminance contrast based on stimulation of the double cones.

**Figure 9:**
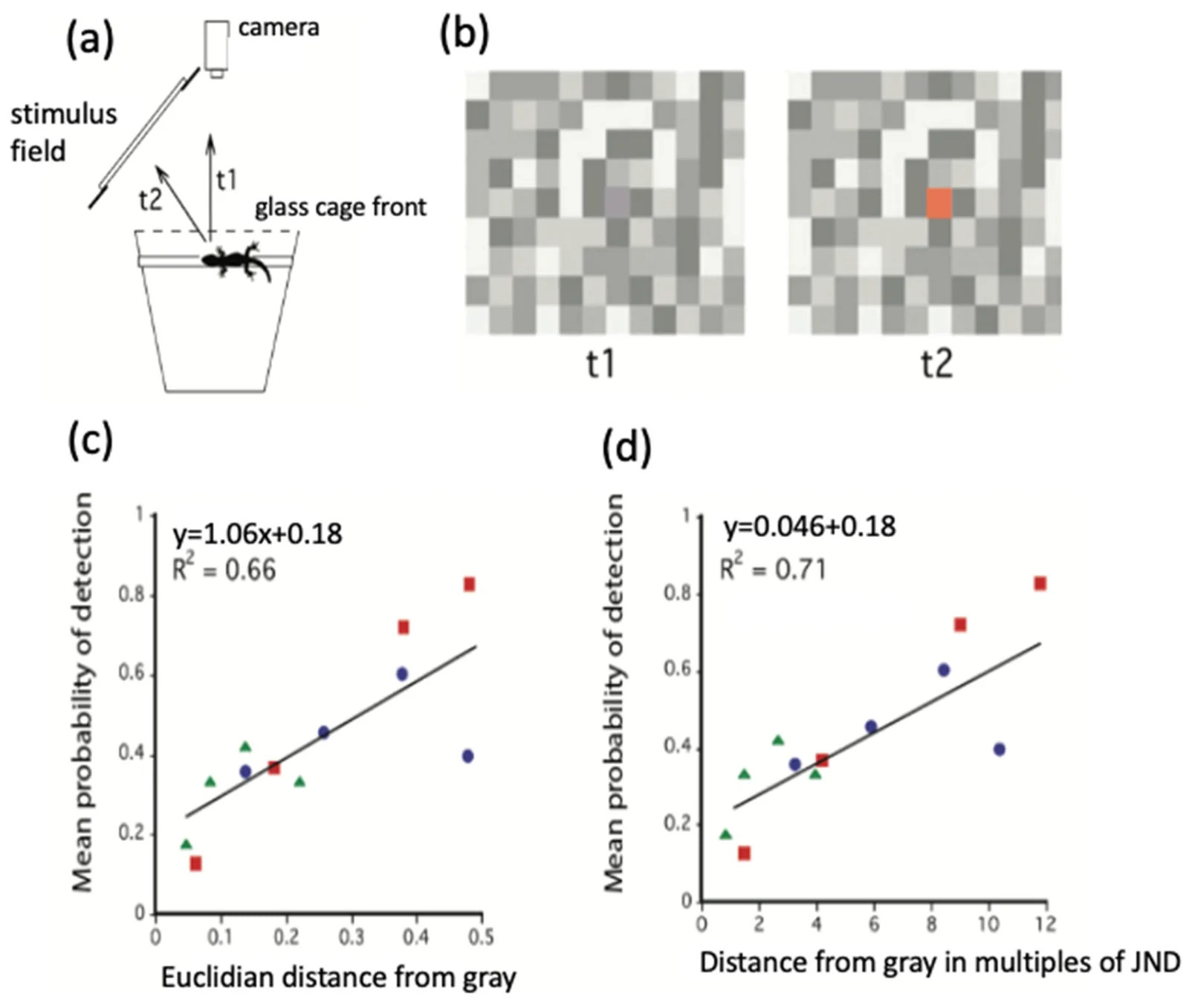
(**a**). Set-up of experiments from [50]. (**b**). The background consists of a checkerboard pattern of gray squares that vary in luminance. The center square has a median luminance. At the onset of a trial, the lizard is perched parallel to the clear front of the cage with its gaze directly outward (time = t1). Eye position is monitored with a video camera. In a trial, a central square of the background is abruptly replaced with a colored stimulus square of equal luminance. A positive response is recorded if the lizard shifts its gaze abruptly to the introduced color stimulus (=t2). In each experiment, 10 lizards were tested five times for each stimulus condition. Test colors consisted of green, red, or blue squares combined with varying amounts of gray. (**c**,**d**). Linear regression models of response probability vs. distance in perceptual space of the stimulus from the gray background square. Colors of dots, triangles and squares indicate stimulus colors. (**c**). In this case, chromatic contrast was calculated as a distance in a triangular chromatic space. There was no ultraviolet in the stimulus or background, so a triangle replaces the tetrahedron of Figure 7. There was a significant linear correlation between the response probability and chromatic distance. (**d**). An alternative method for quantifying chromatic contrast is to use the RNL model to determine the distance between the background and stimulus square in units of Just Noticeable Difference. This value also shows a significant linear correlation with response probability. Reproduced with permission from L. Fleishman et al., Behav. Ecol. Sociobiol.; published by Springer, 2016.

Fleishman et al. [50] carried out another experiment, similar to that described above, with *Anolis sagrei*, that focused on the effects of chromatic contrast. In this case, the visible background area consisted of a checkerboard pattern of gray squares of different luminance values (see Figure 9). In a trial in this set of experiments, one of the central gray squares was rapidly replaced with a colored square of the same estimated luminance, but different spectrum. No attempt was made to assess the impact of luminance contrast. Three colors were tested as stimuli: green, blue, and red. For each color, different amounts of gray were added to create a series of stimuli that differed spectrally from the gray background to a different extent. The perceptual distance of the stimulus from the gray background was systematically varied for three different colors, but the average luminance of the background, as well as the luminance of the test square and stimulus, was kept constant.

As in the previous experiment, response probability increased linearly with distance in color space from the background for all three test colors. In this case, two different models were used to calculate an estimated distance in perceptual color space between stimulus and background. First, a Euclidean distance in a triangular space (a color triangle was used rather than a color tetrahedron, because this experiment did not employ any ultraviolet wavelengths). Second, a commonly used model of perceptual color distance, known as a Receptor Noise Limited (RNL) model [52,53,54] was used to estimate perceived chromatic contrast. The RNL model is based on the assumption that color perception is based on the relative stimulation of a set of cones, and differences in perceived colors are based on changes in the relative stimulation of the set of cones. However, as cone pigments absorb photons and create neural signals, there is inherent variability in the response, which creates some noise in the signal. If two color stimuli are very similar, the noise in the color perception channels may make them indistinguishable. When two stimulus colors are just different enough so that photoreceptor channel noise does not render them indistinguishable, they are said to be 1 “just noticeable difference” (JND) apart in perceptual space.

Color stimuli that are more distant in color space can be described based on how many multiples of JND they are apart. It has been demonstrated that the perceptual distance in units of JND yields an effective approximation of how different two colored stimuli appear to a viewer [55,56]. Thus, the distance in units of JND between two color patterns provides an estimate of their distance in perceptual space. This value can be calculated based on the stimulus spectrum and the size and density of the photoreceptor channels that contribute to color vision. As will be described below, both Euclidean distance in color space and the color space based on distance in JND values between colors provide good estimates of behaviorally measured chromatic contrast. There are two advantages to the RNL model: (1) it can be used to estimate when two colors are not distinguishable (the discrimination threshold), and (2) a version of the model has been developed that takes into account the decreased signal-to-noise ratio that occurs under low light [53,54]. 

For this set of experiments, linear least squares regression was used to test the relationship between response probability and signal/background chromatic perceptual distance. Both models yielded strong positive relationships. The R^2^ value for the Euclidean distance in the color triangular space model was 0.66. R^2^ for detection probability versus distance in units of JND in the RNL model was 0.71. The difference between the correlations in the two models was not significant (*p* > 0.05).

This pair of experiments described above demonstrated that contrasts in color and luminance between stimuli and backgrounds strongly influence stimulus detection probability, and the magnitude of these contrast effects can be predicted with a knowledge of the difference in relative stimulation of the four main classes of single cones, and the difference in stimulation of the LWS double cones (i.e., luminance contrast).

### 8.2. Light Intensity/Color Detection Interaction

The two experiments described above were based on an assumption, often made in studies of color perception, that as long as the stimulus intensity is great enough to elicit a response from cones, color perception is independent of total light intensity. However, it is known that at very low light levels, cone-based color perception is lost. In humans, as light intensity approaches the low-intensity range of cone-based perception, color vision is still possible, but the ability to distinguish different colors becomes reduced [57,58,59]. Anoles rely exclusively on cones. They also have small eyes with small pupils that limits the quantity of light that can enter the eye (ca. 1 mm pupil diameter), and small-diameter photoreceptors. This means that under low-light daylight conditions, photon capture rates from visible stimuli become greatly reduced. At low light levels, an additional source of photoreceptor noise becomes important: random fluctuations in the quantity of photons reaching each photoreceptor. This has the effect of decreasing the signal-to-noise ratio within the cones. A reduction in chromatic distance between colored stimuli occurs as the signal-to-noise ratio becomes smaller, and this results in reduced color discrimination capability. Fortunately, there is a version of the RNL model that takes the effects of low light intensities into account [53].

**Figure 10:**
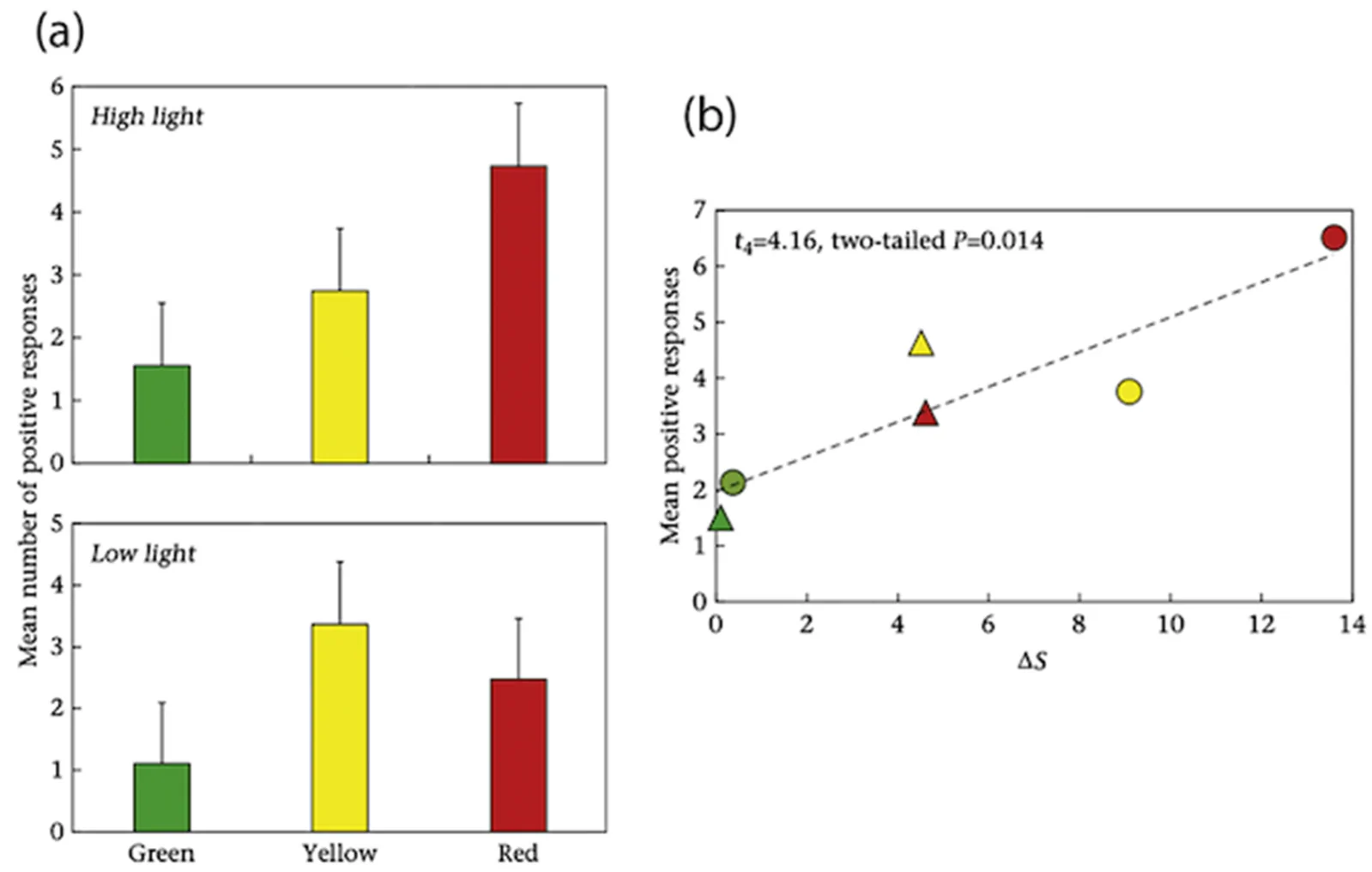
The effects of the interaction between light intensity and chromatic contrast in determination of response probability. In this set of experiments, the background consisted of green squares with gray added to produce a range of different radiance values [51]. In each trial, one of the center squares was replaced by a colored stimulus of nearly equal luminance. A green control stimulus square had a slightly different luminance. The three different stimulus types (green control, yellow, red) were tested in relatively low light (equivalent to forest shade) and relatively high light (equivalent to a low-shade habitat). Each lizard was presented with 8 trials of each stimulus condition, and 11 lizards were tested. (**a**). In the high-light conditions, the red stimulus triggered the most positive responses. In the low-light conditions, the yellow stimulus response changed very little, but response to the red stimulus was significantly reduced. There was a significant interaction (*p* < 0.002) between illumination intensity and stimulus color. (**b**). The results are plotted against calculated chromatic contrast based on a version of the RNL model that includes the effects of low light in units of JND. Circles indicate high light intensity. Triangles indicate low light intensity. Reproduced with permission from L. Fleishman et al., Animal Behaviour; published by Elsevier, 2020.

Fleishman et al. [51] used methods similar to those described above (based on the visual grasp reflex) to see if changes in light intensity impact the relative visibility of different color stimuli in *Anolis sagrei*. These experiments utilized a setup like that shown in Figure 9. In this case, the visual background consisted of a checkerboard composed of green squares that varied in luminance. In each experimental trial, a central green square was replaced with either (1) a green control (differing slightly in luminance, but not in spectrum, from the square it replaced), a yellow square, or a red square. In the latter two cases, the luminance of the background square that was replaced matched that of the color square introduced. The red and yellow squares had the same peak intensity of their spectrum, but the yellow square was, overall, higher in luminance because it reflected more of the spectrum. The background and stimulus squares were printed on card stock, and the experimental area, including the background and stimulus, was illuminated with a broad-spectrum halogen light source. The experiments were carried out at two different light intensities: one equivalent to a heavily shaded forest (=low light, irradiance = 1.2, SD = 0.10 µmol m^−2^ s^−1^) and one equivalent to a mostly unshaded habitat (=high light, irradiance = 275, SD = 25 µmol m^−2^ s^−1^). The results of the experiments are summarized in Figure 10.

Under high light intensity, the red stimulus elicited the highest number of positive responses, and the RNL model showed that it generated the highest chromatic contrast with a green background. When light intensity was low, the response to the red stimulus dropped significantly, and the yellow stimulus elicited a higher number of positive responses than the red. The RNL model-based estimates of detectability of the different color-background combinations (Figure 10b) accurately predicted the shift in visibility of the red stimulus from higher than yellow to lower with the change in light intensity from high to low. Thus, the detection frequency for a colored stimulus against a green background is highest for a red stimulus in intense light, but shifts to a yellow stimulus under lower light intensity.

## 9. Color Vision and Dewlap Coloration

In addition to exploring the relationship between habitat light and eye design, visual ecology involves exploring the relationship between visual system properties and important visual tasks. Anoles communicate with motion patterns of the head and body and the display of a colorful, expandable dewlap. Dewlap colors vary widely among species. The colors of the dewlap of the majority of species cover most of the organ uniformly. A few species have two colors (but one usually covers most of the area) and a smaller number have more complex patterns [60]. Dewlap colors often differ distinctly between sister taxa that inhabit physically adjacent or even overlapping microhabitats. It has been hypothesized that the evolution of distinctly different dewlap colors played an important role in the process of speciation among anoles [5]. This leads to a key question: what evolutionary forces have driven the evolution of distinct dewlap color patterns in different species or populations? The “sensory drive hypothesis” Endler [61] posits that differences in environmental light conditions and/or sensory system features favor different colors or patterns for highest visibility.

The most common use of the dewlap by male anoles is a behavior known as an assertion display [62]. An active adult male will move around its territory spontaneously, producing a display consisting of motion patterns of the head and body and expansion (and sometimes movement) of the dewlap [63]. This display appears to serve the functions of making other males in the area aware that a territory is occupied, attracting females toward the territory, and potentially stimulating females to mate. Macedonia et al. [64] used a robotic lizard, *Anolis grahami*, and showed that dewlap color is an important species identification cue. Anoles are small, and most live in complex vegetated habitats. Selection should favor a dewlap coloration that attracts the visual attention of conspecifics and is easy to see and quickly recognize. It has therefore been hypothesized that each species should evolve a dewlap color that is effective at eliciting attention in its own habitat light conditions. We then ask, do differences in habitat light conditions favor the evolution of among-species differences in dewlap coloration?

It is a fairly straightforward task to estimate the relative visibility of different dewlaps in a given location in the habitat. Animals are captured, and the spectral reflectance and transmittance of the dewlap are measured in the lab with a portable spectroradiometer. Transmittance is important to measure because dewlaps are thin and diffusely transmit light [33]. One can then go into the field and observe locations where lizards are displaying (which they do frequently and spontaneously). The observer then moves to the display location and measures spectral irradiance in two directions in order to quantify the light that would strike a dewlap from front and back relative to a viewer. Then the radiance of the background behind the lizard is measured. With the irradiance data, one can estimate the luminance and spectral radiance of any lab-measured dewlap at the display location. One can then calculate the luminance and chromatic contrast of the dewlap versus the background at this field location. This allows a comparison of the relative detectability of different dewlaps in different habitats. If visual-system response acts as a selective force on dewlap coloration, the dewlap of a species should be more visible in its home habitat light conditions than the dewlaps of other species from other habitats. Note that it is important in this process to measure light at the location where the lizard displays its dewlap because it might be selective in locations where it displays.

Leal and Fleishman [65] compared dewlap colors from four geographically distinct populations of *A. cristatellus* on Puerto Rico that occupied two distinctly different habitat types (mesic vs. xeric forest types). They found that differences in dewlap color resulted in higher luminance contrast for each population in its own habitat. This result was confirmed with field-based behavioral experiments by Gunderson et al. [66]. No significant differences in chromatic contrast were detected.

Fleishman et al. [67] compared dewlap detectability of four Puerto Rican species from habitats that differed strongly in shade level (see Figure 2). They predicted that each species in its natural home habitat should be more visible than the other three species. Instead, they found that the red dewlap of one of the species (*A. pulchellus*) was the most visible in all four habitats. Further, the rank of dewlap visibility of the four species did not change under the light conditions of the four different habitats. In a similar study, Macedonia et al. [68] found a lack of evidence for sensory drive among five Jamaican species, with the same species having the most visible dewlap in all habitats. These outcomes were the result of two phenomena. In all of the habitats, background radiance consisted of bright (sun spots, highly reflective leaves, etc.) and dark (shadows, dark trunks, soil) patches. Darker dewlaps (e.g., red coloration) created high negative luminance contrast with bright background patches, and low contrast with dark patches. Yellow and/or white dewlaps created high positive contrast with dark patches, and low contrast with light patches. Overall, the average luminance contrasts were similar for the different colored dewlaps. When spectral quality was assessed, the background in all of the habitats was dominated by green color (of vegetation), against which red consistently created the highest chromatic contrast. These results suggested that red or orange should nearly always be the most common dewlap color, since it stands out most strongly against the green color that dominates most backgrounds. However, Nicholson et al. [60] examined dewlap colors in 140 species and found yellow to be the most common dewlap color.

These previous two analyses [67,68] relied on the results of [43] to predict the visibility of different colors. That study, however, did not account for the effects of low levels of light intensity on chromatic contrast. In a more recent study, Fleishman et al. [51] demonstrated that against a green background, a red stimulus creates a very high chromatic contrast. However, under low-light conditions, the visibility of the red is greatly reduced, and a higher radiance stimulus (yellow, for example) becomes relatively more detectable. The receptor noise model that accounts for a reduced signal-to-noise ratio under low-light conditions [53,54] accurately predicted these light-intensity-based changes in chromatic contrast.

With this additional information, Fleishman et al. [7] revisited the relationship between habitat light and dewlap color in a study of 17 species from Jamaica, Puerto Rico, and the Dominican Republic. The dewlaps of these species range in color from white to yellow to orange to red.

**Figure 11:**
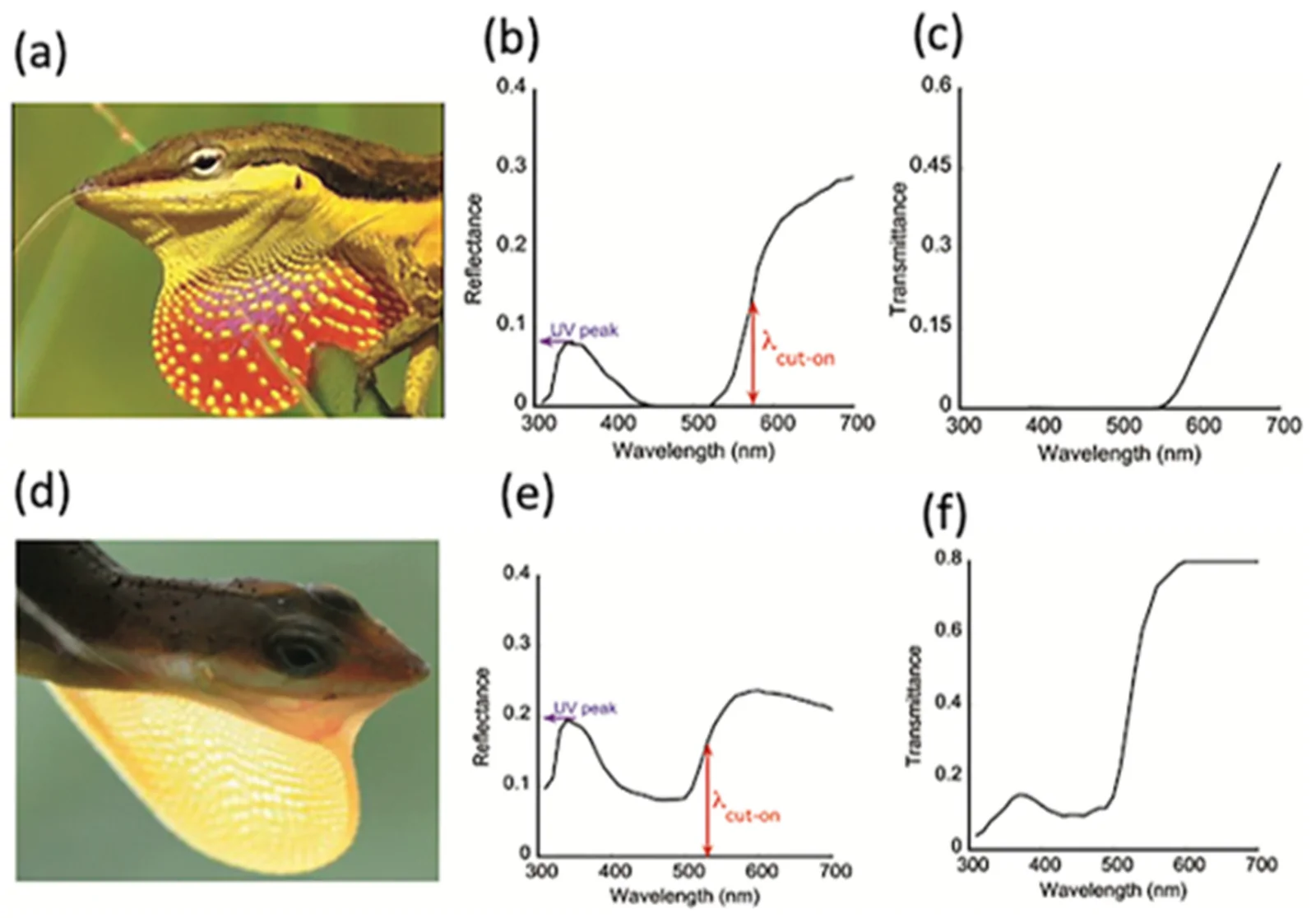
Examples of dewlap color of two sister species. (**a**). The expanded dewlap of *Anolis pulchellus*, a grass anole from Puerto Rico that occupies unshaded habitat. (**b**). The spectral reflectance of the *pulchellus* dewlap. The wavelength at which the reflectance is halfway between minimum and maximum is called λ_cut-on_. The further λ_cut-on_ is to the right, the “redder” the dewlap appears. (**c**). The spectral transmittance of the *pulchellus* dewlap. Dewlaps are thin, and transmitted light contributes to their color appearance. (**d**). The dewlap of *A. krugi*, the sister species of *pulchellus*, which occupies partially shaded forest edge and small gap habitats. The spectral reflectance (**e**) and transmittance (**f**) of the *krugi* dewlap. Photographs by L. Fleishman and M. Leal. Reproduced with permission from L. Fleishman et al., The American Naturalist; published by U. Chicago Press, 2022.

Examples of dewlap reflectance and transmittance are shown for two of these species in Figure 11. The reflectance curves of all of the species in this study have a characteristic shape. Starting at the short-wavelength end of the spectrum, dewlaps of some species exhibit some ultraviolet reflection. This is followed by a low reflectance region in the middle of the curve. The reflectance then rises steeply to a longer-wavelength plateau. The wavelength at which the rising reflectance curve reaches 50% of its maximum is referred to as the “cut-on wavelength” (λ_cut-on_). The appearance of the dewlap to a human observer (e.g., yellow versus red) depends on the value of λ_cut-on_. The further to the right (i.e., toward longer wavelength) the cut-on wavelength is, the redder the dewlap appears.

**Figure 12:**
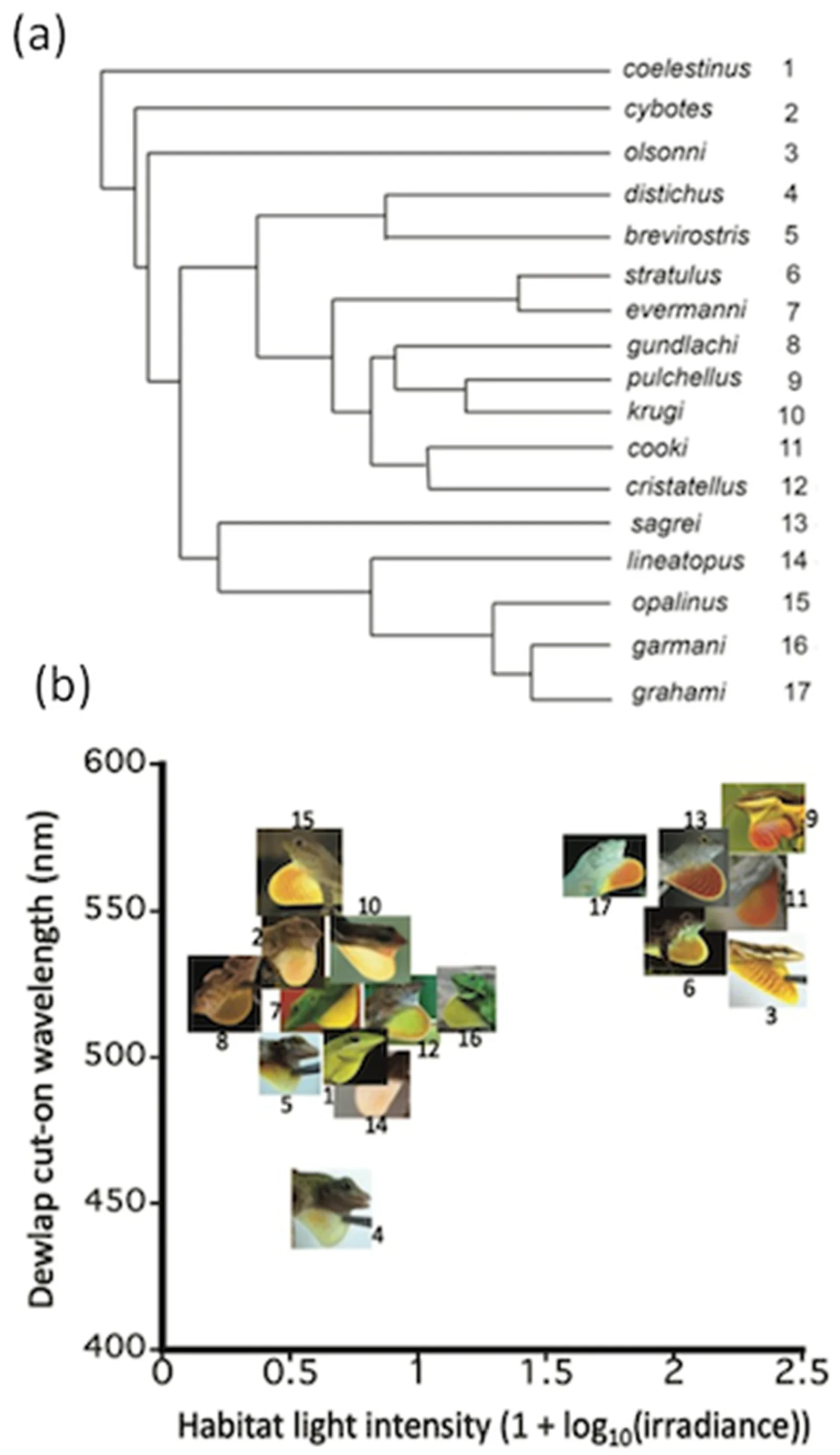
Habitat light and dewlap spectra for 17 species from the Dominican Republic, Jamaica and Puerto Rico. (**a**). A pruned phylogenetic tree is shown. Numbers refer to the photographs below. (**b**). A plot of λ_cut-on_ versus mean habitat light intensity with each species represented photographically. Photographs by L. Fleishman and M. Leal. Reproduced with permission from L. Fleishman et al., The American Naturalist; published by U. Chicago Press, 2022.

The full set of species examined in this study is shown in Figure 12a. Figure 12b is a graph of λ_cut-on_ versus average habitat irradiance. It is evident that lizards from high-intensity habitats have orange or red dewlap colors and a longer λ_cut-on_. Species from more shaded, darker habitats are more variable in color, but typically have white or yellow dewlaps, associated with a shorter λ_cut-on_. Using phylogenetic generalized least squares models [69] it was found that there is a significant positive correlation between habitat light intensity and λ_cut-on_ (*p* = 0.023, t = 2.54, df =15) among this set of species [7]. In general, dewlaps occupying more intensely lit habitats possess “redder” dewlaps.

In order to assess the reason for the relationship between habitat light intensity and red versus yellow dewlaps, Fleishman et al. [7] carried out detailed comparisons of three species pairs. In each case, the members of the pair were sister species or very closely related. Each pair included one species with a yellow dewlap and one with a red dewlap. Each species was observed in its own habitat, and light conditions were quantified (spectral irradiance and background spectral radiance). At each measurement location, the luminance and chromatic contrasts between the dewlap and the natural background were calculated for the dewlaps of both species. It was hypothesized that if dewlap visibility in different habitat light conditions favored the evolution of the observed differences in dewlap color, then in each location the “home” species dewlap should be more visible than the “non-home” sister species.

**Figure 13:**
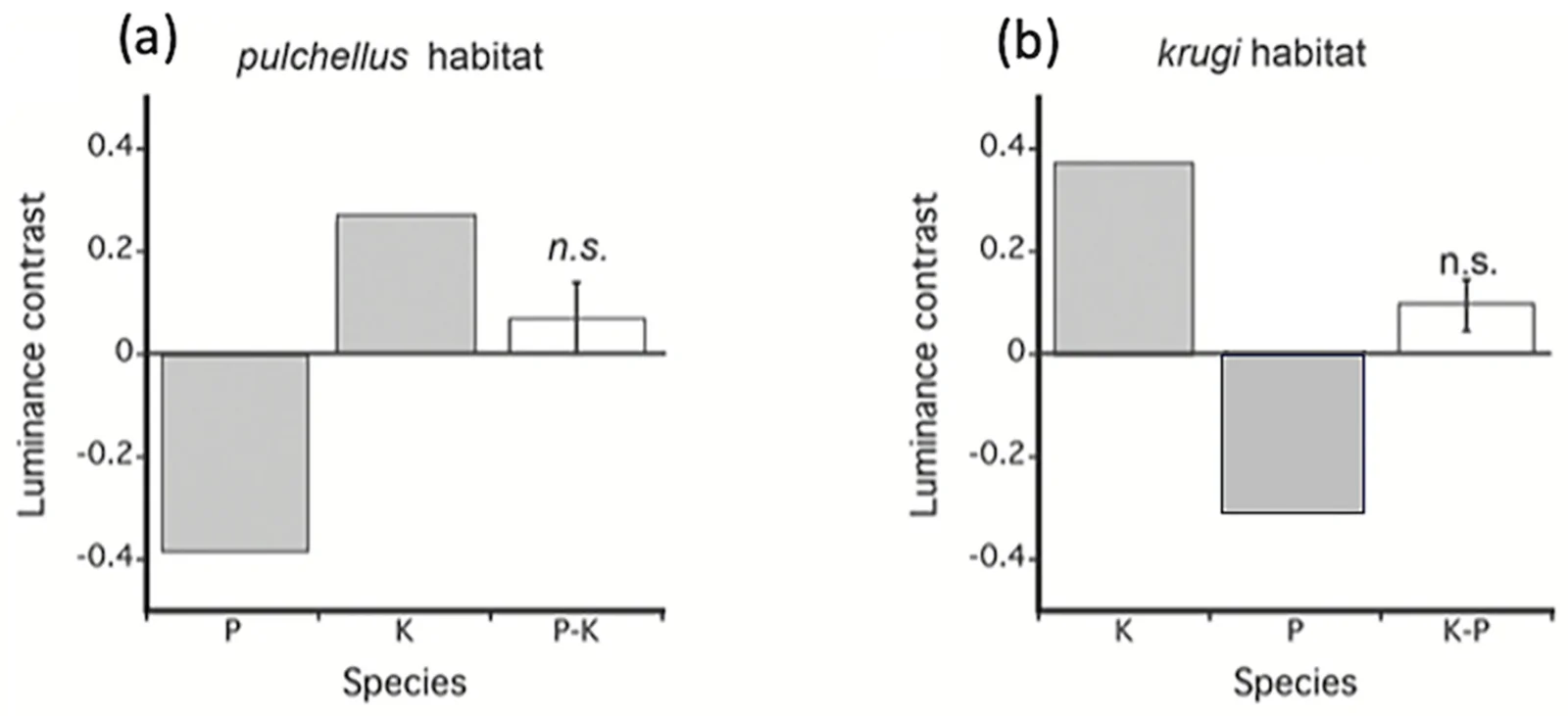
Mean luminance contrast for (**a**) *A. pulchellus* and (**b**) *A. krugi* calculated for display sites of both species in their natural habitat. The species that is found in a given habitat is referred to as the “home” species. At each site, the values for both species (home and non-home) were determined (shaded bars). Negative contrast indicated that the dewlap was usually darker than the background. The unshaded bars indicate the mean difference between the absolute values of contrast of the two species at each site. In each case, the value from the non-home species is subtracted from the value for the home species. The letters (P, K) indicate which habitat the measurements come from. Error bars are the standard error of the mean. Significance levels (n.s. = not significant) refer to the results of *t*-tests comparing the contrast magnitude difference at each site to 0. Reproduced with permission from L. Fleishman et al., The American Naturalist; published by U. Chicago Press, 2022.

Here the results are described for the pair of sister species shown in Figure 12. *Anolis pulchellus* occupies unshaded grassy areas, and *A. krugi* occupies partially shaded grass/bush habitats. Light measurements were taken at each site where they were observed displaying. Luminance and chromatic contrast were calculated for both species. Figure 13 shows the average luminance contrast for the two species in each habitat, as well as the difference between the two species in the absolute value of luminance contrast (home species minus the other species), which was statistically compared to zero. No significant difference from zero was found, indicating that the dewlap colors have not diverged in a way that creates greater luminance contrast in each habitat. In this example (and other species-pair comparisons), it was consistently found that the absolute value of luminance contrast differed little between dark and light dewlaps.

**Figure 14:**
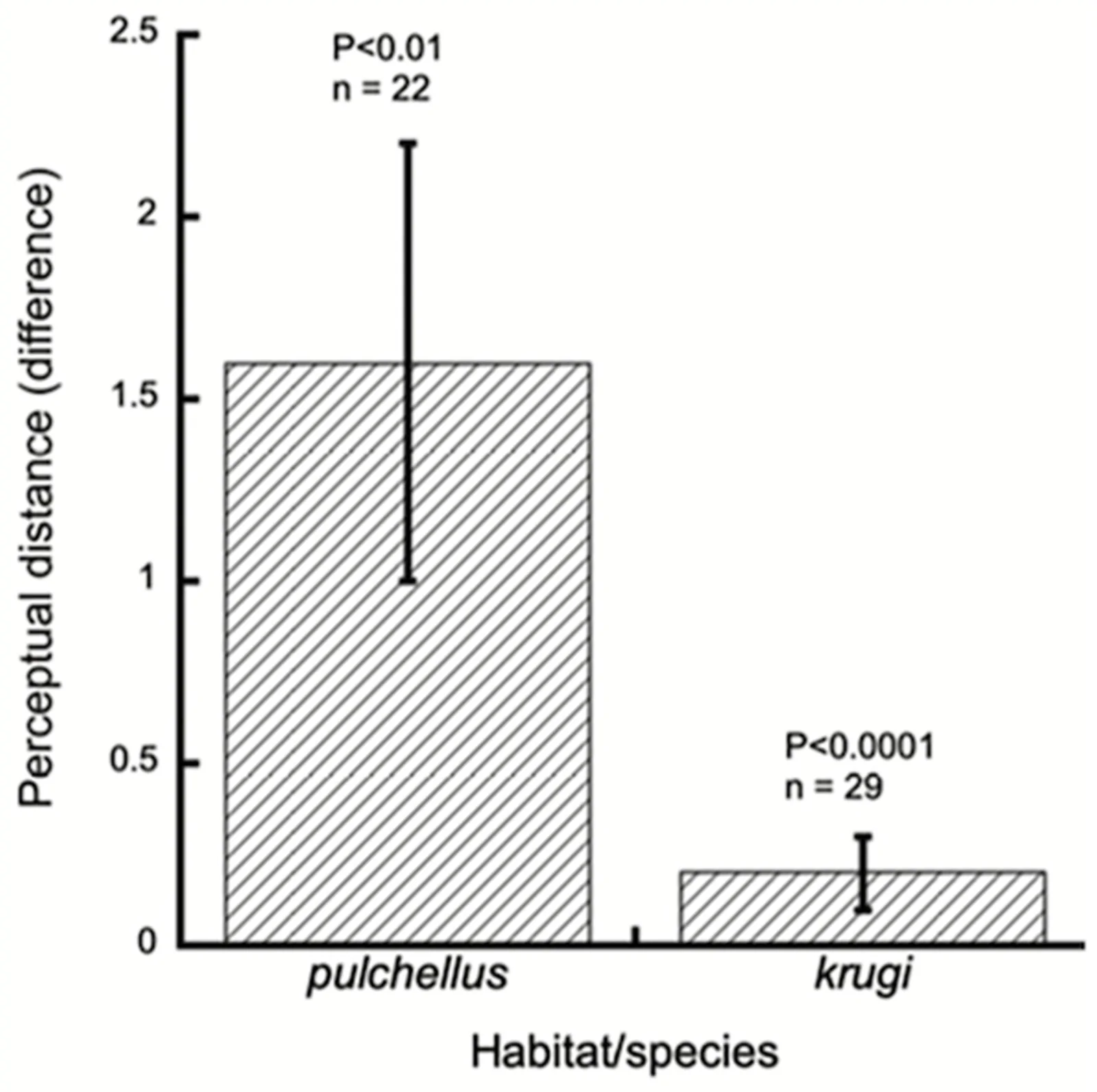
The difference in mean chromatic contrast (based on RNL modeling of JND difference between dewlap and background) of two species measured in the habitat of each: *A. pulchellus* (grass species, low shade, high light intensity), *A. krugi* (grass species, forest edge, moderately low light intensity). The measurements of light were made in the habitat of the species listed on the x-axis. Chromatic contrast was calculated for the home species and the non-home sister species at locations where individuals of the home species were observed. At each location, chromatic contrast (dewlap vs. background) was calculated for both species. The value for the non-home species was then subtracted from the value for the home species. If the home species is more visible, the difference is expected to be greater than zero at most locations. For each species of the pair, it was tested whether these difference values were significantly greater than zero (*t*-test). In both cases, the home species showed significantly higher chromatic contrast. The results for this pair of species are typical. Two other closely related species pairs (low-shade vs. high-shade habitat) were tested; the same results were obtained. Error bars indicate the standard error of the mean. Reproduced with permission from L. Fleishman et al., The American Naturalist; published by U. Chicago Press, 2022.

Next comparisons were made of chromatic contrast using the RNL model with effects of light intensity included [19,53,54]. Results are summarized in Figure 14. In the brightly lit habitat of *A. pulchellus*, its red dewlap consistently exhibited higher chromatic contrast than the *krugi* dewlap. However, in the shadier habitat of *A. krugi*, its bright yellow dewlap created a significantly higher chromatic contrast than the red dewlap of *pulchellus*. These results were replicated with two other species pairs. Under low-moderate light, yellow dewlap colors create a greater chromatic contrast against the natural green background. Under the bright light conditions of a low-shade habitat, an orange or red dewlap creates a much greater chromatic contrast. Fleishman et al. [7] thus concluded that the interaction of light intensity and chromatic contrast is responsible for the pattern observed in Figure 12. It is worth noting that one of the species pairs—*A. pulchellus* and *A. krugi*—were compared in the study described earlier [67]. In that case, *A. pulchellus* was found to be the most visible in all habitats. The difference is that in the latter study (Figure 13 and Figure 14), the effects of low light intensity on chromatic contrast were taken into account.

This relationship has been noted in other papers: dewlaps of lizards from brighter habitats are often reddish in color, while those from darker habitats are most often yellow or white [63,70]. These results are consistent with the behavioral experiments that showed that a red stimulus against a green background is most effective in high light, but a yellow stimulus (which reflects more total photon flux) creates a greater chromatic contrast against a green background in relatively low light. This phenomenon appears to occur because anoles, which possess small eyes and pupils, begin to see the effects of low-light noise on chromatic contrast under moderately low-light conditions.

Another feature of dewlap coloration that relates to the visual system is the presence of ultraviolet coloration in many dewlaps. After UV visual capability was discovered in anoles [71] scientists began measuring anoline dewlaps to look for UV coloration. It was soon determined that many species, but not all, possess this trait. Fleishman et al. [7] found no correlation between habitat light conditions and ultraviolet dewlap reflectance. They also found that the clade of species that inhabit Jamaica all lack UV reflectance, suggesting a phylogenetic constraint. However, a close relative of these species, *A. conspersus* from Grand Cayman, has the highest dewlap UV reflectance reported for any anole [72]. The factors that explain the presence or absence of UV reflectance of the dewlap remain unclear.

Presence or absence of ultraviolet coloration appears to play an important role in species recognition in some cases. For example, two species—*A. cooki* and *A. cristatellus*—occupy partially overlapping habitat in southwestern Puerto Rico. The species are very similar in overall appearance, and their dewlaps appear nearly identical to a human observer. It turns out, however, that the *A. cristatellus* dewlap is ultraviolet reflective and the dewlap of *A.cooki* is not, which makes the two dewlaps easy for the species to tell apart [73].

## 10. Motion Vision

Another important aspect of anoline visual ecology is the perception of visual motion. *Anolis*, like most lizard species, rely extensively on visual perception of motion [63]. Most of their food consists of moving prey. They detect the presence of predators by their movement. Their communication (mate attraction, aggressive interactions, territory control) relies heavily on patterns of movement of the head, body, and dewlap [6,74]. Understanding the visual ecology of motion perception requires answers to a few basic questions: (1) How strongly do lizards respond to different patterns of visual motion? (2) How have motion perception properties of the visual system influenced the evolution of motion patterns utilized by anoles in their communication system? (3) How do variables such as habitat motion noise caused by windblown vegetation or distance to an intended signal receiver impact perception and properties of moving displays?

The response of individual neurons to motion stimuli has not been studied directly in any anoline species. Studies have been carried out in the green iguana (*Iguana iguana*), which is in the same subfamily and shares basic neuroanatomy with the anoles. These studies examined the response of single neurons in the optic tectum, the brain region responsible for directing attention to relevant locations and for motion processing. It was found that responses of iguanas were similar to those of mammals [75]. This suggests that the neural basis for motion processing is, to a large extent, evolutionarily conservative across the vertebrates, which allows us to assume that motion responses of *Iguana iguana* are likely to be similar in the anoles [75,76].

Results from these iguana studies offer some clues about visual motion perception in *Anolis*. Many of the motion-sensitive cells exhibited a directionally selective response and fell into three velocity-tuned categories: high, middle, and low. The receptive fields of the motion-sensitive cells mapped to different regions of visual space, were circular or elliptical in shape, and were larger with distance from the fovea. Tectal neurons typically responded with a strong burst of increased activity to motion onsets and offsets or to a flash of light. Most of the cells habituated rapidly to a moving stimulus, but regained sensitivity after 20–30 s. These results suggest that three attributes of visually moving stimuli are critical: the location of the moving stimulus in visual space, the direction of movement, and angular speed across the retina. Because of their directional selectivity and their tendency to fire strongly at the onset of motion, populations of tectal cells are well-designed to encode the timing of changes in motion direction and respond strongly to visual stimuluss motion stops and starts. The detection of continuously directional stimuli depends on the velocity (in degrees of visual angle per unit time). For stimuli that stop and start and reverse their direction of movement, with each shift in stimulus direction, a new population of cells will fire in unison, precisely marking the timing of these transitions. Lizard visual displays exhibit these properties as the head and body move up and down.

While there are no published studies of single-neuron responses to motion in *Anolis*, tectal-evoked potential responses, based on recordings from large numbers of neurons at once, were carried out by Persons et al. [77]. The results were largely consistent with those observed in iguanas. Strong tectal potentials were evoked by stimulus motion onset. The potentials showed rapid habituation. Response amplitudes were directly proportional to the stimulus-versus-background luminance contrast.

There have been several studies of anoline motion perception at the behavioral level that have relied on the visual grasp reflex to test relative detectability of different motion stimuli. Motion stimuli were presented in the visual periphery, and the probability of a shift of gaze (and attention) to the different motion stimuli was recorded. Fleishman [63,78] studied *A. auratus*. A small stimulus lure attached via a thin thread to an electronically controlled motor was positioned in the visual periphery. It was moved up and down (linear y-axis motion) in different temporal patterns. In these experiments, in each stimulus trial, the amplitude of the up-and-down motion started very small and was steadily increased until a response was recorded. Surprisingly, the motion amplitude at which a response occurred was nearly the same across all stimulus patterns: a visual angle of vertical motion of 0.2–0.4 degrees. If this amplitude was reached and passed without response, there was usually no response at all. Although responses to different patterns tended to occur at the same amplitude, the probability of any positive response varied significantly with stimulus motion pattern. Different stimulus motion patterns tested included sinusoidal motion from 0.5 to 10 Hz, 1.5 Hz square wave (abrupt up-and-down shifts), and different combinations of acceleration and velocity. By far the most effective stimulus was the low-frequency (e.g., 1.5 Hz) square-wave motion: a stimulus consisting of very high acceleration to high velocity (which was very similar to the square wave). In other words, motion patterns that involved abrupt jumps from one position to another up and down elicited the most responses.

Fleishman [78] also studied the effect of visual noise in the form of movement of windblown vegetation. Either prior to or during the behavioral tests with the small moving bead, an artificial background of plastic plants was rocked up and down in a sinusoidal motion pattern resembling the motion of windblown vegetation. When the stimulus motion was similar in frequency and waveform to the background plant motion, response probability was significantly reduced. When the square wave pattern was used for the stimulus (and sinusoidal motion for the plants), the plant motion did not significantly reduce the response. These results suggest two ways in which the anoline motion perception system deals with the visual noise of vegetation movement. First, it is most responsive to abrupt start-and-stop movements and is less responsive to the roughly sinusoidal and continuous rocking motion of windblown vegetation. Second, in the presence of windblown vegetation, the motion vision habituates quickly to the commonly present patterns and frequencies of motion. Taken together, these results suggest that the highest visibility motion pattern is an abrupt square-wave-like movement, and this pattern maintains its effectiveness in the presence of windblown vegetation.

Consistent with these results, Fleishman [79] discovered that a common predator of anoles in Panama—the vine snake *Oxybelis aeneus*—moves preferentially when windblown vegetation is in motion. In addition, the snake adds a plant-mimicking back-and-forth rocking movement to its forward locomotion. It appears that this predator of small vertebrates has evolved a stalking behavior that takes advantage of the anoline motion perception system.

The result that a motion amplitude of approximately 0.4 degrees of visual angle is the strongest motion stimulus has been supported by a number of other experiments in which lizards were presented with abrupt up-and-down motion stimuli in the visual periphery, at a series of different amplitudes, and the probability of a gaze shift toward the stimulus was recorded. Five different species have been tested in this way, and in all cases the peak response occurred when the movement stimuli were between 0.2 and 0.8 degrees of visual angle [26,80,81].

### Motion Patterns as Communication Signals

Nearly all species of anoles rely on motion patterns for communication signals. These include up-and-down motion of the head, “push-up” displays created by movement of the upper body by flexion of the front arms, and expansion and retraction of the dewlap. It is an interesting visual ecology question to consider how the visual-system perception of motion has impacted the evolution of motion patterns as signals. The displays can vary from simple nodding motions of the head alone to complex combinations of the different display elements through time [74]. Less commonly, some other elements are added, such as swishing of the tail, waving of the arm, or a back-and-forth rocking motion.

There is considerable variety across species in the details of these motion displays. Some species have simple nodding patterns that are not highly stereotyped, while others have multiple elaborate, highly stereotyped patterns of head and dewlap. Among the species that have been studied most closely, certain patterns emerge. In most species, a pattern of motion is observed in a variety of display contexts that consists of a species-specific, highly stereotyped temporal pattern of distinct up-and-down head and/or body movements known as the “signature display”. This pattern appears to transmit information about species identity. There are also examples where the signature differs among individuals, and in these cases the signature bob may also signal individual identity [74].

Displays largely occur in three main contexts. Both males and females produce signals related to courtship and/or courtship rejection. Adult males move around their territory and frequently produce a display that lacks aggressive modifiers (see below). This is referred to as an assertion display [62,74]. Its functions appear to be to repel competing males from entering the territory, and to attract and stimulate potential mates. The assertion display is typically directed at conspecific individuals a fair distance away (e.g., 1 m or more). However, the display is also produced in the absence of other individuals within sight [82,83]. The third context is referred to as a “challenge” display [62] which is produced in agonistic contexts to other males at close range. In territorial disputes, male lizards will alternate producing the challenge displays. These encounters usually end with one male fleeing or the two males engaging in a physical fight. Female lizards will also display—in response to male courtship signals (rejection or acceptance) or in agonistic interactions with other females [84]. The signature display pattern typically appears in both challenge and assertion display contexts, and in some species during the courtship display.

There are three likely sources of information contained within these displays: temporal pattern, amplitude variations, and static modifiers. The distinctive temporal pattern of up-and-down movement transmits reliable information about species, and possibly individual, identity. The use of a stereotyped pattern of timed up-and-down movements is consistent with motion perception mechanisms that are highly responsive to direction reversals of visual stimuli, and the brain can therefore record these temporal patterns reliably. The message is the same even if the displaying individual is partly obscured since the full body moves up and down. As long as the movements are above a minimum detection threshold, the pattern can be reliably detected at any distance. Macedonia et al. [64] used robotic lizards to demonstrate that the temporal pattern of motion in a display was an important cue for species recognition.

**Figure 15:**
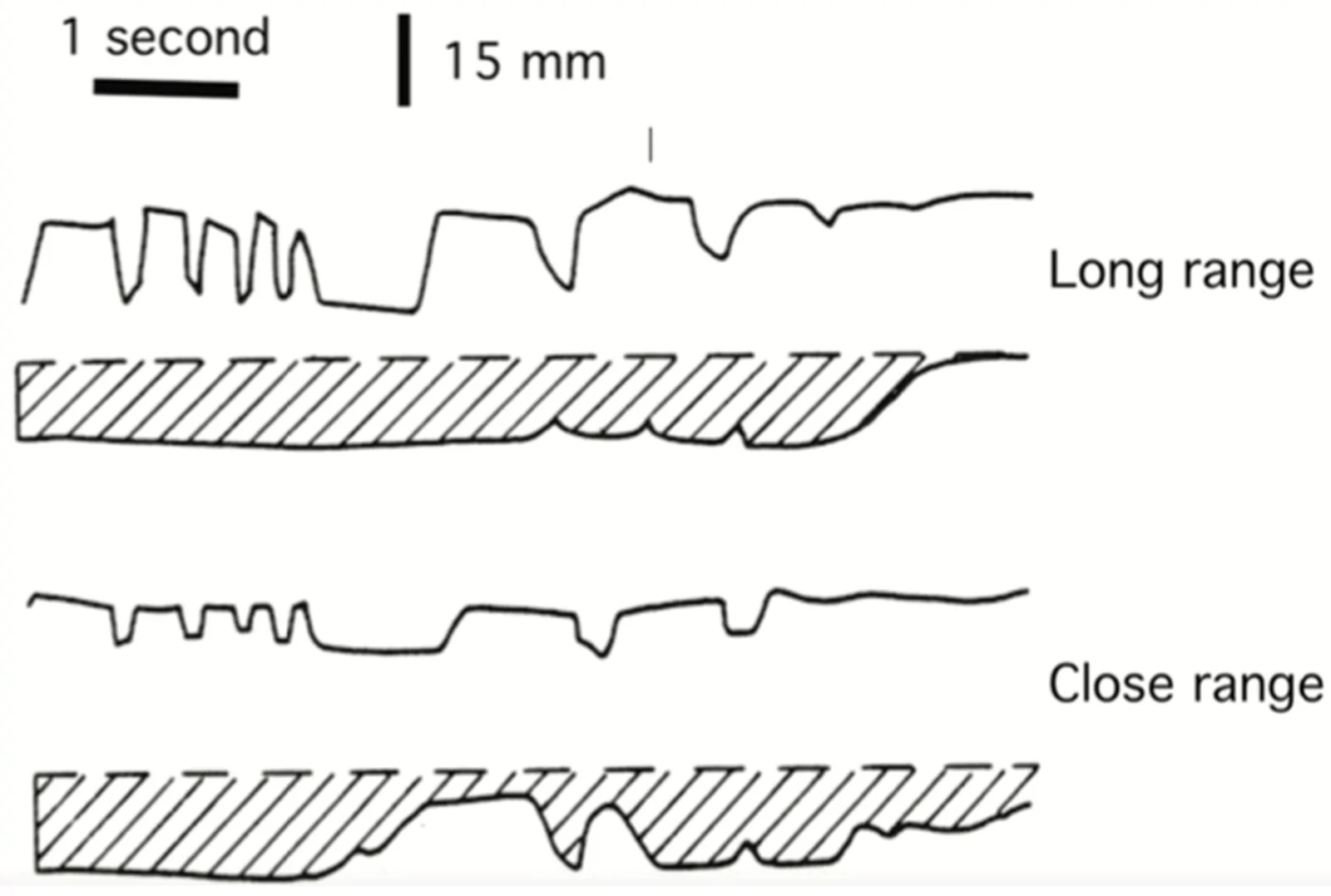
Examples of displays by *Anolis auratus* from [63]. The dark upper line in each display shows the position of the eye along the axis of greatest motion over time. The lower hatched area shows dewlap extension below the chin. The upper display is an example of an assertion display, recorded in the field with no other lizards in view. The lower display is a challenge display, triggered by placing an adult male next to the displaying male. Reproduced with permission from L. Fleishman et al., The American Naturalist; published by U. Chicago Press, 1992.

It has been shown in several species that the amplitude of some of the display movements will vary. These amplitude variations are probably not reliable sources of information. The view of the displaying animal may be partly obscured. Assessing amplitude variations requires the capacity to precisely judge distance to the signaler. Rather than transmit information, it appears that display amplitude variability is a mechanism used by signalers to improve the display as a visual stimulus [85]. Fleishman [63,78] compared displays of adult male *A. auratus* given spontaneously by territorial males in the absence of nearby receivers to displays presented at close range to introduced male intruders. Samples of these two display types are shown in Figure 15. It is apparent that in the long-distance context the initial portion of the display is significantly increased in amplitude, but the timing of motion shifts is unchanged. The introductory portion of the display thus consists of abrupt square-wave-like motions. It was concluded that these motion patterns strongly stimulate the visual periphery of individuals some distance away. When signaling to close-range intruders, the large amplitude motions are not necessary. Thus, the change in display amplitude improves the likelihood of the signal being detected, but does not carry information per se.

Other examples of amplitude variation as a mechanism to improve visibility have been reported. Ord and Stamps [85] recorded spontaneous displays of *A. gundlachi* in natural habitat. They noted that the signature display was sometimes, but not always, preceded by a series of square-wave-like motion patterns—a pattern that would be predicted to increase the likelihood of detection. They also found that the frequency of inclusion of these introductory movements increased with the presence of windblown vegetation and under low-light conditions. They further demonstrated, with the use of a robotic lizard, that the addition of these square-wave-like movements to the beginning of the display increased the rate of detection by nearby viewers. These relatively high-amplitude, abrupt introductory movements are believed to serve as an “alerting” signal that elicits visual attention so an intended receiver will be more likely to see the information contained in the more complex display movements that follow. Several other Puerto Rican species have also been observed to introduce their assertion displays with a series of square-wave-like movements [86,87]. Stamps and Barlow [88] observed a similar alerting signal by *A. aeneus*, used when a signal receiver was far away.

Steinberg and Leal [26] measured displays by *A. gundlachi* in response to introduced intruders at various distances (up to 3 m). Under these conditions, the introductory square wave movements were always observed. However, the amplitude of these movements varied inversely with distance to the intruder. They showed, with motion stimulus experiments using the visual grasp reflex, that stop-start motion patterns of 0.2 to 0.8 degrees of visual angle produced the greatest probability of detection. They found that display motion amplitudes were modified depending on the distance to the intruder, so that they almost always produced motion amplitudes of 0.2–0.8 degrees as seen by the signal recipient. Not only did motion amplitudes increase as intruders were further away, but the amplitudes of the display components were also smaller when the intruders were closer. In this way, the display introductory movements provided nearly optimal motion stimuli for detection by the intruder.

Ord et al. [89] report another form of motion that appears to effectively elicit attention. Unlike the Puerto Rican species that tend to utilize introductory square-wave-like head movements as “alerting signals”, the species from the Jamaican clade use a rapid opening of the dewlap (without head movement) for a similar function. While the motion is not as abrupt as in the head movements described earlier, the combination of a rapid movement of the leading edge of the dewlap and the abrupt appearance of a distinct color makes it an effective motion pattern.

Another sort of information delivered by displays is graded indications of motivational state. This occurs, for example, in agonistic interactions. Most of this information is conveyed by static modifiers that accompany the threat displays, such as lateral flattening of the body, mouth opening, or extension of the nuchal crest [74]. These are not motion patterns, but accompany the threat displays. One case in which graded signaling is accomplished by motion patterns is reported for *A. limifrons*. These lizards have five different display patterns which indicate different levels of aggressive motivation in fighting interactions [90]. This is a somewhat unusual case of different temporal patterns used as graded displays.

## 11. Discussion

The main questions that are asked in studies of the visual ecology of an animal group are: (1) What aspects of visual perception are widely shared across most of the species and thus represent the ancestral condition? (2) What are the habitat light conditions in which different species operate, and how do they differ? (3) Have the eyes of species in the group evolved away from shared ancestral traits in response to differences in light conditions? If not, then why not? (4) To what extent do the differences among species in habitat light and visual perception result in differences in their behavior and/or physical form?

Broadly speaking, the answers to these questions for *Anolis* are as follows. (1) The anatomy and physiology of the components of the visual systems of the different species are very similar. One exception is the red-shifted visual pigments of *A. carolinensis*. (2) While the habitat light conditions of a relatively small proportion of anoline species have been studied, a few basic patterns have emerged. The typical background spectrum in most anoline habitats is dominated by green vegetation. This background radiance tends to select for an achromatic luminance channel that peaks in the green region of the spectrum. The most important difference among habitats in terms of visual function is the large differences in total light intensity that result from different levels of shade or canopy position. (3) The visual systems of the different anoline species have largely retained shared inherited patterns that have changed only modestly over evolutionary time. The similarity of the visual pigments of the retina to what is seen in birds suggests that this pattern was formed early in the rise of terrestrial vertebrates and has seen relatively little change. It is not clear why there has been so little evolutionary diversification of the features of anoline eyes. The same pigment genes are found in many fishes, and these have changed extensively in response to habitat light differences. This suggests that there is no mechanistic factor preventing change. It seems, rather, that the distribution of visual pigments found in anoles—and the similar pattern found in other reptiles and diurnal birds is highly effective in a wide range of habitats, and has been maintained through stabilizing selection. (4) We examined perception of visual displays as an important visual task. While perception of color and brightness is fairly similar for most anoles, the different habitat light conditions select for differences in display behavior. In low light intensity conditions, a more reflective (and transmissive) dewlap is required in order to be visible and recognizable. Thus, we often see yellow or white dewlaps in darker habitats. In high light intensity, dewlap colors tend toward the maximum contrast against the green vegetation background, often leading to red or orange dewlaps. Thus, the visual signal properties are not selected for by differences in visual system properties, but by the way in which a shared visual system operates under different light conditions.

The visual motion perception properties select for certain patterns in visual display. When signal detection is more difficult—for example, under windy conditions or when receivers are far away, lizards utilize patterns that most effectively stimulate motion circuits. Thus, in the presence of windblown vegetation, when signal receivers are not close by, anoles will utilize abrupt high-amplitude movements. There is no evidence of differences among the species in their response to motion. As with color, it is habitat conditions that create the need for different patterns of signaling behavior.

## 12. Conclusions

With its large number of species from diverse habitats, well-established phylogeny, and heavy reliance on vision and visual communication, the genus *Anolis* continues to be an important model system for studies of sensory system evolution.

Studies thus far reveal several obvious questions that can be studied in the future. Examples include analysis of the role of the temporal fovea in prey capture, and more generally, analysis of depth perception. The functions and effects of photoreceptor oil droplets and their variations are largely a mystery.

Several species are emerging as useful laboratory subjects. For example, *Anolis sagrei* is a common invasive species (in Florida and elsewhere) that can be easily collected without impacting any natural population, and is easy to care for. Areas of interest for new research include studies of their perception of depth and distance and studies focusing on the role of retinal oil droplets in visual adaptation. New techniques in color analysis, including ways to utilize video and the availability of multi-spectral imaging systems, offer many new possibilities for studying the connections between habitat light, signal function and dewlap coloration and color pattern [91].

*Anolis* is rapidly emerging as a model system in a number of other new areas. Their genome is available, and genetic manipulation methods such as CRISPR have been developed [92]. A number of labs are using anoles to study the development of visual systems (for example, [93,94,95]. Another area of great promise is the study of the different processing areas of the brain in relation to the ecological and behavioral complexity of different species (e.g., [96]).

Just as their diversity and fascinating behavior have allowed the analysis of the processes of visual-system and visual-signal evolution, the group represents an appealing model system that will, without doubt, be attractive to future scientists studying a variety of topics related to visual processing and brain evolution.

## Figures and Tables

**Table 1:** Descriptions of oil droplets found in 17 *Anolis* species (From [15]).

Description	Abbreviation	50% λ_cut-on_ * (Average nm)
C1	colorless	378
C2	colorless	340 **
G	green	503
Y	yellow	467

* Oil droplets absorb almost all short-wavelength light, then shift abruptly to nearly 100% transmission. λ_cut-on_ is the wavelength at which the shift from minimum to maximum transmission is halfway. ** Measurements were not made for wavelengths shorter than 350 nm. This value is estimated based on the fact that C2 was associated with UVS cones.

## Data Availability

No new data were created or analyzed in this study. Data sharing is not applicable to this article.

## Abbreviations

The following abbreviations are used in this manuscript:

CFF	Critical frequency of fusion
MSP	Microspectrophotometry
LWS	Long-wavelength-sensitive
MWS	Middle-wavelength-sensitive
SWS	Short-wavelength-sensitive
UVS	Ultraviolet-sensitive
LS	Stimulus luminance
LB	Background luminance
CL	Luminance contrast
CC	Chromatic contrast
RNL	Receptor noise limited
JND	Just noticeable difference
UV	Ultraviolet
ERG	Electroretinogram
